# New zinc(ii) metalloporphyrin: molecular structure, spectroscopic characterization, electrochemical sensing of dopamine, and catalytic dye degradation†

**DOI:** 10.1039/d5ra00762c

**Published:** 2025-04-01

**Authors:** Mohamed Achraf Bouicha, Chama Mabrouk, Bouzid Gassoumi, Houcine Barhoumi, Florian Molton, Frédérique Loiseau, Thierry Roisnel, Aracely Serrano Medina, Jose Manuel Cornejo Bravo, Eduardo Alberto Lopez-Maldonado, Habib Nasri

**Affiliations:** a University of Monastir, Laboratory of Physical Chemistry of Materials (LR01ES19), Faculty of Sciences of Monastir Avenue de l'environnement 5019 Monastir Tunisia Habib.Nasri@fsm.rnu.tn; b University of Monastir, Laboratory of Interfaces and Advanced Materials, Faculty of Sciences of Monastir Avenue de l'environnement 5019 Monastir Tunisia; c Département de Chimie Moléculaire, Université Grenoble Alpes 301 rue de la Chimie, CS 40700 38058 Grenoble Cedex 9 France; d Institute of Chemical Sciences of Rennes, UMR 6226 University of Rennes 1, Beaulieu Campus 35042 Rennes France; e Facultad de Medicina y Psicología, Universidad Autónoma de Baja California Tijuana 22390 Mexico; f Faculty of Chemical Sciences and Engineering, Autonomous University of Baja California 22424 Mexico elopez92@uabc.edu.mx +216 73 500 278

## Abstract

This work is a continuation of the series of studies aimed at studying the electronic and structural properties of divalent metal porphyrin complexes, especially zinc(ii) metalloporphyrins. In this perspective, we have prepared the [Zn(TMPP)(4,4′-bpy)]·CHCl_3_ (I) coordination compound, where TMPP is the *meso*-tetra(*para*-methoxyphenyl)porphyrinate and 4,4′-bpy is the 4,4′-bipyridine. The UV/Vis, fluorescence, IR and ^1^H NMR spectroscopic techniques, ESI-HRMS mass spectrometry investigation as well as a single crystal X-ray diffraction study were used to characterize the title compound. Notably, we demonstrated the ability of this zinc(ii) metalloporphyrin to degrade the methylene blue (MB) dye, examining several influencing factors, including pH, temperature and initial dye concentration. Additionally, complex I exhibited remarkable efficiency in degrading MB under blue LED irradiation. Beyond catalytic applications, this compound was successfully employed as an electrochemical sensor for the detection of dopamine (DA) using the square wave voltammetry (SWV) method, showcasing its multifunctional capabilities.

## Abstract 2

This work is a continuation of the series of studies aimed at studying the electronic and structural properties of divalent metal porphyrin complexes, especially zinc(ii) metalloporphyrins.

## Graphical abstract

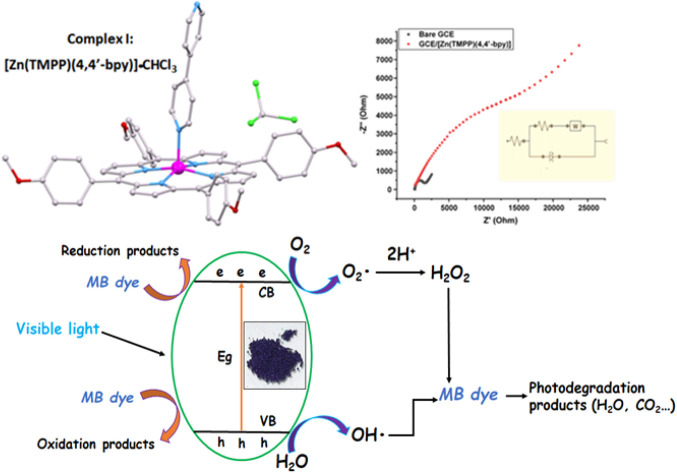

## 1 Introduction

Since the pioneering work of Hans Fischer in the early 20th century, metalloporphyrins have captivated the attention of researchers worldwide due to their intriguing electronic, magnetic, and catalytic properties. Among these, zinc(ii) porphyrin complexes stand out as a cornerstone in the field, owing to their unique structural features and diverse applications.

The most used metalloporphyrins as models for hemoproteins such as hemoglobin, myoglobin, and cytochromes P450 are iron porphyrin complexes because of the presence of the iron as a center ion in hemoproteins. Just to have an idea about the most investigated metals in porphyrin complexes, we used the Cambridge Structural Database updated to November 2023 (ref. 1) to classify the reported crystal structures of porphyrin complexes for several metal ions.

According to Table S1,† the most important reported metalloporphyrin crystal structures are those with zinc(ii) metal ion. These porphyrin complexes are among the most studied metalloporphyrins due to several key reasons:

(1) The insertion of Zn(ii) ion into the porphyrin cavity is very easy compared to the insertion of other divalent M(ii) center metals by using the zinc acetate common synthetic route.^2^ The zinc(ii) metallation leads to very stable metalloporphyrins.

(2) Zn(ii) metalloporphyrins provide much simpler coordination compounds than those of cobalt, iron or other d transition metals to study the influence of different types of axial ligands on the physical chemistry properties of metal porphyrins. Indeed, the zinc metal ion is unambiguously in the II oxidation state.

(3) Zinc(ii) porphyrins exhibit unique photophysical properties due to their closed-shell properties that make them attractive for various applications. They have strong absorption in the visible region, high fluorescence quantum yields, and long-lived excited states.^3^

Despite the electrochemical inactivity of Zn(ii) due to its fully filled d-orbitals, Zn(ii) porphyrin complexes can still participate in redox processes. This is made possible through the delocalized π-electron system of the porphyrin and its interactions with axial ligands, which enhance electron transfer. Consequently, Zn(ii) porphyrins have proven valuable for electrochemical sensing applications.

Moreover, various molecular electrocatalysts have been widely explored for detecting glucose, pesticides, and pharmaceutical compounds, demonstrating broad applicability in electroanalysis.^4,5^ These findings underscore the versatility of metal porphyrin systems, not only in sensing but also in more complex catalytic applications. In this context, a review highlighted the use of indium tin oxide electrodes modified with materials such as ZnO nanowire arrays and graphene foam for the electrochemical determination of l-Dopa, a drug molecule structurally similar to dopamine.^6^

Furthermore, small molecular d^10^ metal complexes, particularly Zn-based systems, have attracted considerable interest in electrocatalysis due to their unique electronic properties and various applications, including water splitting and sensing technologies.^7–10^ Although these complexes are electrochemically inactive in their pure metal form, they exhibit catalytic activity when their electronic structure is modified through ligand interactions. This behavior allows them to facilitate reactions such as oxygen and hydrogen evolution while also enhancing their efficiency in sensor applications. These findings highlight the broad potential of d^10^ metal systems in advancing energy and environmental technologies.

On the other hand, 4,4′-bipyridine is a heterocyclic compound widely used in various materials and compounds.^11^

**Scheme 1:**
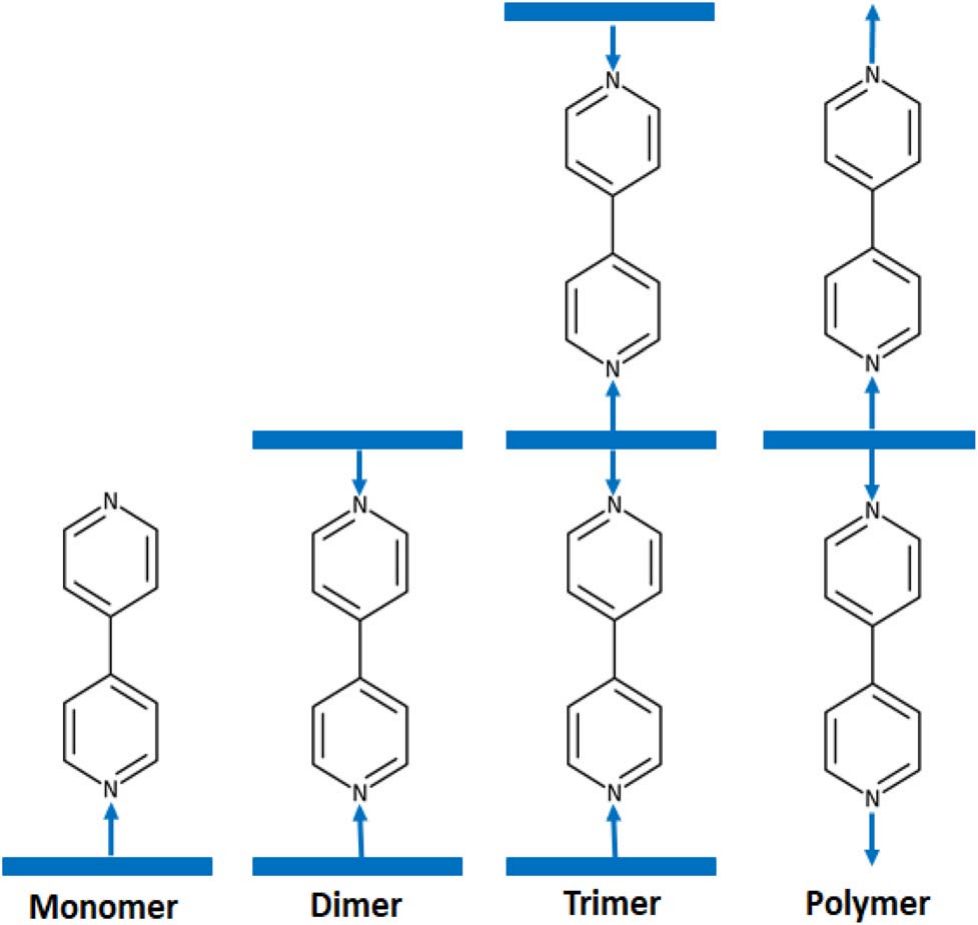
Schematic illustrations of the various structural types of zinc porphyrins with 4,4′-bipyridine axial ligand.

4,4′-Bipyridine and its derivatives have numerous applications in chemistry and materials science. They are used as ligands in coordination chemistry. These compounds are widely used as ligands in coordination chemistry as demonstrated by the very important number of molecular structures of 4,4′-bpy-metal complexes reported in the CCDC Cambridge database (more than 680 hits) (Cambridge Structural Database updated to November 2023). Among 4,4′-bpy metal complexes, 4,4′-bpy metalloporphyrins present an important class of coordination compounds including a large variety of center ions such as Fe(ii), Fe(iii), Ru(ii), Os(ii), Ni(ii), Mn(iii), V(iv) and mainly Zn(ii). Several investigations of these zinc(ii) metalloporphyrins have been reported especially this last decade.^12–15^ It is noteworthy that in solid state 4,4′-bpy zinc(ii) metalloporphyrins crystallized as monomers such as [Zn(TEBOP)(4,4′-bpy)] (TEBOP = *meso*-(tetraethyl-4(4-butyryl)oxyphenyl)porphyrinate),^16^ dimers such as [{Zn(TPP)}_2_(μ_2_-4,4′-bpy)] (TPP = *meso*-tetraphenylporphyrinate),^17^ trimers such as [{Zn(TPP)}_3_(μ_2_-4,4′-bpy)]^15^ and as polymers such as {[Zn(TMP)(4,4′-bpy)]}_*n*_ (TMP = *meso*-tetramesitylporphyrinate)^18^(Scheme 1).

The widespread use of organic dyes, such as methylene blue (MB), presents a significant environmental challenge due to their resistance to conventional degradation methods.^19,20^ As a result, there is a growing need for eco-friendly and efficient strategies to degrade these dyes. Oxidative and photocatalytic degradation methods^19^ have emerged as promising solutions to address water contamination caused by toxic pollutants. Synthetic metalloporphyrins, particularly Zn(ii) porphyrins, have gained attention as effective photocatalysts in such degradation processes. These catalysts utilize reactive oxygen species (ROS) to break down contaminants in a manner similar to peroxidase-like catalytic activity. For instance, the degradation of methylene blue (MB) using Zn(ii) porphyrin in the presence of H_2_O_2_ involves the generation of hydroxyl radicals (˙OH), which accelerates the breakdown of the dye. This mechanism relates on hydroxylation and oxidative cleavage pathways, eventually leading to the complete mineralization of the dye molecules.^21^

Recent studies, including those conducted by our research team, have demonstrated the successful degradation of various organic dyes using *meso*-arylporphyrins and metalloporphyrins as catalysts.^13,16,22–25^ Building on this work, we aim to investigate the dual functionality of Zn(ii) porphyrin complexes in both environmental and biomedical applications. Specifically, we focus on the degradation of methylene blue dye and the detection of dopamine (DA), highlighting the redox-active nature of these complexes and their ability to facilitate charge transfer processes in both oxidative and photocatalytic degradation, as well as in electrochemical sensing. This dual functionality, stemming from the ability of Zn(ii) porphyrins to modulate electron density and engage in redox reactions, positions them as promising candidates for both environmental remediation and biosensing technologies.

In this context, we present the synthesis and characterization of the (4,4′-bipyridine)[*meso*-tetra(*para*-methoxyphenyl)porphyrinato]zinc(ii) chloroform monosolvate complex [Zn(TMPP)(4,4′-bpy)]·CHCl_3_ (I). The structure of this complex was determined using UV-Vis, fluorescence, IR, ^1^H NMR, cyclic voltammetry, and ESI-HRMS techniques. The crystal structure was analyzed, and the intermolecular interactions contributing to its stability were studied using surface Hirshfeld analysis. We also investigated the degradation of methylene blue dye through photodegradation and oxidative degradation, using complex I as a catalyst. The activation energy and thermodynamic variables for the oxidative degradation were determined using the pseudo-first-order model. Furthermore, we explored the electrochemical detection of dopamine (DA) in human urine samples using the square wave voltammetry (SWV) technique.

## 2 Experimental section

### 2.1 Synthetic procedures

#### 2.1.1. Synthesis of H_2_TMPP

**Scheme 2:**
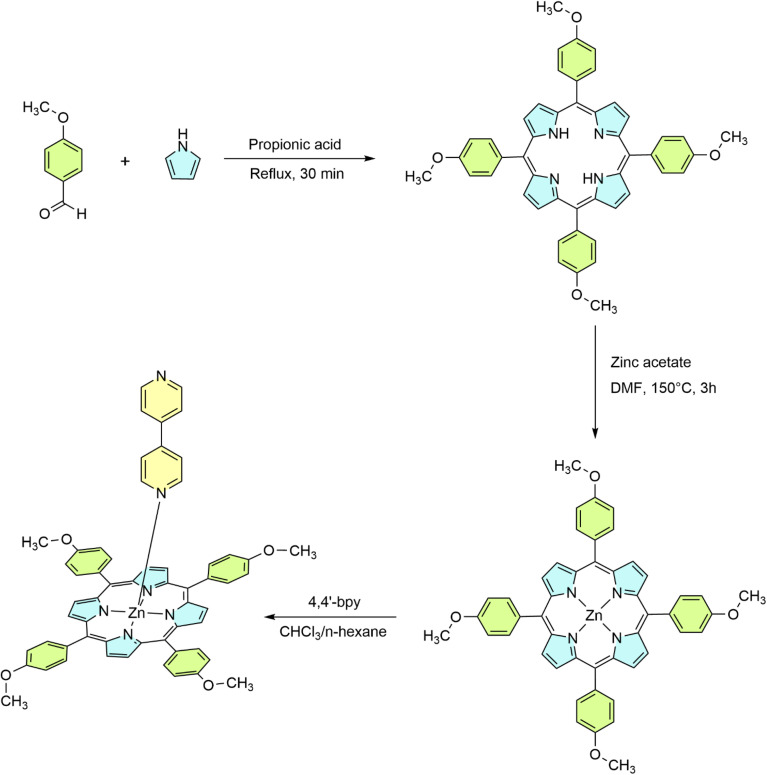
Synthetic pathway for H_2_TMPP, [Zn(TMPP)] and [Zn(TMPP)(4,4′-bpy)]·CHCl_3_ (I).

The synthesis of the H_2_TMPP porphyrin was made according to the Adler and Longo procedure^26^ (Scheme 2). A solution of 4-methoxybenzaldehyde (4.5 g, 0.033 mol) in 85 mL of propionic acid was prepared in a 250 mL two-neck flask equipped with a condenser. The reaction mixture was stirred and maintained under reflux at 140 °C for 30 minutes. Subsequently, 2.5 mL of pyrrole was introduced. After completion, the mixture was left to cool overnight in the dark. The resulting solid was collected and purified by sequential washing with distilled water and *n*-hexane. The final porphyrin product was obtained as a purple powder with a mass of 4.75 g (% yield = 19).

UV-Vis: *λ*_max_ (nm, CH_2_Cl_2_, log *ε*): 423 (6.24), 519 (4.95), 557(4.82), 596 (4.65), 651 (4.70) nm. FT-IR (solid, cm^−1^): 3320 (w) [*ν*(NH) pyrrole], 2990–2839 (m) [*ν*(CH) Porph.], 1243 (s) [*ν*(CO) methoxy], 964 (m) [*δ*(CCH) Porph] ^1^H NMR (400 MHz, CDCl_3_*δ*(ppm)): 8.87 (s, 8H, H_β_), 8.11 (d, 8H, H_o,o′_), 7.29 (d, 8H, H_m_), 4.11 (s, 12H, H_a_), −2.74 (s, 2H, NH_py_).

#### 2.1.2. Synthesis of [Zn(TMPP)]

The metallation of H_2_TMPP with zinc(ii) was carried out using the traditional metal-acetate/dimethylformamide (DMF) method described by Buchler.^2^ In this process, 500 mg (0.68 mmol, 1 equivalent) of *meso*-tetra(*para*-methoxyphenyl)porphyrin was dissolved in 100 mL of DMF and heated to 150 °C. Zinc acetate dihydrate (Zn(CH_3_COO)_2_·2H_2_O) (1.493 g, 6.8 mmol, 10 equivalents) was added to the solution and stirred continuously for 3 hours until the complete conversion of the free-base porphyrin, as monitored by UV-Vis spectroscopy and thin-layer chromatography (TLC) on silica gel using chloroform as the mobile phase.

The reaction mixture was then cooled to 50 °C, and 50 mL of water was added to precipitate the product. The solid obtained was filtered, washed with *n*-hexane, and dried under vacuum for 30 minutes, yielding the [Zn(TMPP)] complex with an 83% yield (450 mg) (Scheme 2).

UV-Vis: *λ*_max_ (nm, CH_2_Cl_2_, log *ε*): 425 (6.36), 551 (5.17) 592 (4.92) nm. FT-IR (solid, cm^−1^): 3000–2825 (m) [*ν*(CH) Porph.], 1264 (s) [*ν*(CO) methoxy], 1001 (m) [*δ*(CCH) Porph] ^1^H NMR (400 MHz, CDCl_3_*δ*(ppm)): 8.95 (s, 8H, H_β_), 8.14 (d, 8H, H_o,o′_), 7.30 (d, 8H, H_m_), 4.10 (s, 12H, H_a_).

#### 2.1.3. Synthesis of [Zn(TMPP)(4,4′-bpy)]·CHCl_3_ (I)

A solution of [Zn(TMPP)] (20 mg, 0.0250 mmol) and 4,4′-bipyridine (60 mg, 0.384 mmol) was prepared in 5 mL of chloroform and stirred at room temperature overnight (12 hours). During the reaction, the mixture underwent a color change from purple to green-blue. Crystals of complex I were obtained by allowing *n*-hexane to diffuse slowly into the chloroform solution (Scheme 2).

UV-Vis: *λ*_max_ (nm, CH_2_Cl_2_, log *ε*): 432 (6.07), 558 (4.96), 604 (4.84) nm. FT-IR (solid, cm^−1^): 3109 (w) [*ν*(CH) bpy], 3033–2834 (m) [*ν*(CH) Porph.], 1507 (s) [*ν*(CN) bpy], 1234 (s) [*ν*(CO) methoxy], 995 (m) [*δ*(CCH) Porph] ^1^H NMR (400 MHz, CDCl_3_*δ*(ppm)): 8.81 (s, 8H, H_β_), 8.01 (d, 8H, H_o,o′_), 7.21 (d, 8H, H_m_), 4.06 (s, 12H, H_a_).

### 2.2 X-ray molecular structure

High-quality crystals of the title compound were grown by the slow diffusion of *n*-hexane into a chloroform solution of Complex I. For the X-ray diffraction analysis, a dark purple prism-shaped single crystal with dimensions of 0.29 × 0.20 × 0.07 mm^3^ was selected. Data collection was performed at 150(2) K using a Bruker AXS D8 VENTURE diffractometer^27^ with Mo Kα radiation (*λ* = 0.71073 Å), and the structure was solved using direct methods *via* the SIR-2014 program^28^ and refined through full-matrix least-squares techniques on *F*^2^ with the SHELXL-2014 program.^29^ Hydrogen atoms were incorporated into the refinement using the riding model. Intermolecular interactions were analyzed using the PLATON program,^30^ and packing diagrams were generated with MERCURY software.^31^ Crystallographic and structural parameters are summarized in Table 1.

**Table 1:** Crystal data and structural refinement details for [Zn(TMPP)(4,4′-bpy)]·CHCl_3_ (I)

Formula	C_59_H_45_Cl_3_N_6_O_4_Zn
M.W.	1073.73
Crystal system	Monoclinic
Crystal	*P*2_1_/*c*
*a* (Å)	13.0739(14)
*b* (Å)	14.9439(16)
*c* (Å)	11.6472(12)
*α* (°)	90
*β* (°)	98.352(4)
*γ* (°)	90
*V* (Å^3^)	4972.2(9)
*Z*	4
*ρ*_calc._/g cm^−3^	1.434
*μ*/mm^−1^	0.712
*F*(000)	2216
Crystal size (mm^3^)	0.29 × 0.20 × 0.07
Crystal color	Violet
Crystal shape	Prism
*T* (K)	150 (2)
*θ*_min_ – *θ*_max_ (°)	1.872–27.488
Limiting indices	−16 ≤ *h* ≤ 16, −42 ≤ *k* ≤ 38, −15 ≤ *l* ≤ 15
*R* (int)	0.0209
Total/unique data	68 934/11 367
Observed data [*F*_o_ > 4*σ*(*F*_o_)]	10 090
Parameters/rest	658/0
*S* [goodness of fit]	1.021
*R*_1_ a, w*R*_2_^b^ [*F*_o_ > 4*σ*(*F*_o_)]	*R*_1_ = 0.0415; w*R*_2_ = 0.1066
*R*_1_ a,w*R*_2_^b^ [all data]	*R*_1_ = 0, 0.0471; w*R*_2_ = 0.1103
Min./max. Res. (e Å^−3^)	0.899/−0.737
CCDC	2 358 252

a *R*_1_ = Σ‖*F*_o_|–|*F*_c_‖/Σ|*F*_o_|.

b w*R*_2_ = {Σ[w(|*F*_o_|^2^–|*F*_c_|^2^)^2^]/Σ[w(|*F*_o_|^2^)^2^]}^1/2^.

## 3 Results and discussion

### 3.1 Mass spectrometry

The [Zn(TMPP)] starting material and complex I were characterized using high-resolution mass spectrometry ESI, as depicted in Fig. S1 and S2,† respectively. In Fig. S2,† fragments corresponding to [Zn(TMPP) + H]^+^ and [Zn(TMPP)(4,4′-bpy) + H]^+^ are observed with *m*/*z* values of 797.2119 and 953.2794, respectively. These values are consistent with the theoretical *m*/*z* values of 797.2101 and 953.2700. These results from high-resolution ESI mass spectrometry confirm the stability of the [Zn(TMPP)(4,4′-bpy)]·CHCl_3_ (I) complex in dichloromethane solution.

### 3.2 ^1^H NMR and IR spectroscopy

The ^1^H NMR spectra of the H_2_TMPP and the [Zn(TMPP)] complex, recorded in CDCl_3_, are shown in Fig. S3 and S4.† In the spectrum of H_2_TMPP, the NH-pyrrolic inner protons exhibit strong shielding, appearing at a chemical shift of −2.74 ppm. The β-pyrrolic protons and aromatic phenyl protons (H_o,o′_ and H_m,m′_) resonate within the range of 8.87 to 7.29 ppm. A singlet at 4.11 ppm corresponds to the methoxy protons. For [Zn(TMPP)], the disappearance of the −2.74 ppm signal unequivocally confirms the insertion of the Zn(ii) ion into the porphyrin core. The β-pyrrolic and phenyl protons in [Zn(TMPP)] exhibit slight shifts compared to those in the free-base H_2_TMPP, reflecting the diamagnetic nature of the [Zn(TMPP)] complex and the subtle electronic changes caused by metallation.

The ^1^H NMR spectrum of [Zn(TMPP)(4,4′-bpy)]·CHCl_3_ (I) which is shown in Fig. S5† is characteristic of a diamagnetic zinc(ii) *meso*-arylporphyrin complex. The chemical shift values of the β pyrrolic, H_o_,_o′_, H_m_,_m′_ and H(OCH_3_) protons of the TMPP porphyrinate are 8.81, 8.01, 7.21 and 4.06 ppm, respectively (Table S2†). The spectrum further reveals distinct signals corresponding to the axial 4,4′-bipyridine ligand. The aromatic protons (C–H) of the 4,4′-bpy ring appear downfield, with chemical shifts observed at 6.17 and 5.57 ppm. These protons are significantly more shielded compared to those in the non-coordinated 4,4′-bipyridine molecule, which exhibit chemical shift values of 8.74 and 7.53 ppm, respectively.

The IR spectra of H_2_TMPP, [Zn(TMPP)] and [Zn(TMPP)(4,4′-bpy)]·CHCl_3_ depicted in Fig. S6, S7 and S8,† respectively, were recorded in solid state in the range of [4000–500 cm^−1^].

The IR spectrum of the H_2_TMPP exhibits (Fig. S6†) (i) a weak absorption band at 3320 cm^−1^ attributed to the stretching frequency *ν*(N–H) of the pyrrole rings, (ii) a multiple weak bands between 2990 and 2839 cm^−1^ corresponding to *ν*(C–H) of the TMPP porphyrinate, (iii) a strong absorption band at 1243 cm^−1^ attributed to the *ν*(C–O) stretching frequency of the OMe groups of the TMPP porphyrinate and (iv) a strong band corresponding to the deformation frequency *δ*(CCH) with a wavenumber value of 864 cm^−1^ of the porphyrin macrocycle. Upon the insertion of the zinc(ii) ion into the porphyrin ring ([Zn(TMPP)] complex), the band attributed to the N–H vibration frequency of the free base porphyrin disappears and the band corresponding to *δ*(CCH) of the porphyrin macrocycle is shifted toward the high frequencies (*ν̄* = 999 cm). For this starting material (Fig. S7†), the *ν*(C–H) of the porphyrin core and the *ν*(C–O) stretching frequency of the OMe groups present *ν̄* values very close to those of the H_2_TMPP free base porphyrin. For complex I, the weak absorption band with *ν̄* value of 3108 cm^−1^ and the strong IR band at 1493 cm^−1^ (Fig. S8†) are attributed to the *ν*(C–H) and *ν*(C=N) stretching frequencies of the 4,4′-bpy axial ligand. The characteristic porphyrinate frequencies of complex I resonate at wavenumber values very close to those of H_2_TMPP and [Zn(TMPP)].

### 3.3 UV-visible absorption spectroscopy

**Fig. 1:**
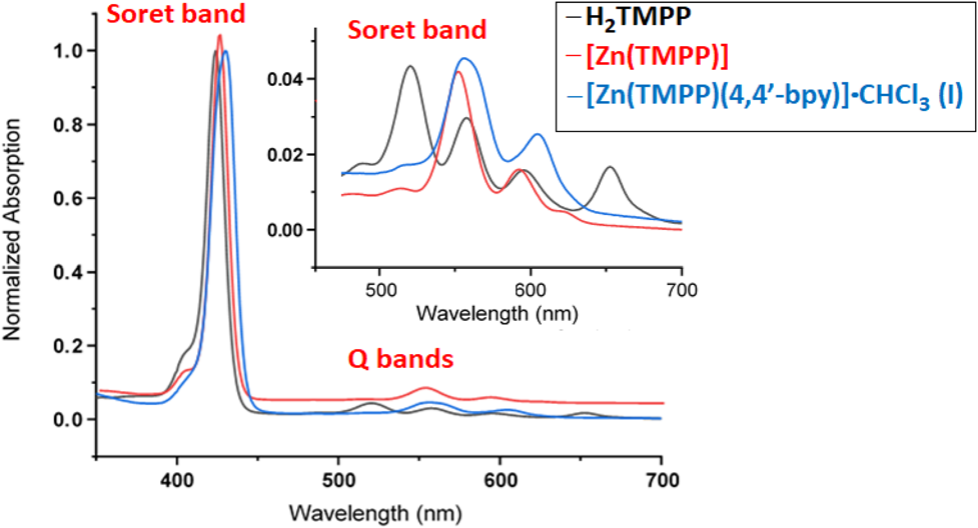
UV-Vis spectra of H_2_TMPP, [Zn(TMPP)] and [Zn(TMPP)(4,4′-bpy)]·CHCl_3_ (I).

The UV-Vis absorption spectra of H_2_TMPP, [Zn(TMPP)] and complex I recorded in dichloromethane solvent are shown in Fig. 1 and in Table 2 are given the *λ*_max_ values of the Soret and Q bands of these three porphyrinic species along with those of a selection of *meso*-arylporphyrins and zinc(ii) metalloporphyrins.

**Table 2:** UV-Vis data of H_2_TMPP, [Zn(TMPP)] and [Zn(TMPP)(4,4′-bpy)]·CHCl_3_ (I) and a selection of *meso*-porphyrins and Zn(ii)-porphyrins complexes

Compound	Solvent	*λ*_max_ (nm)		Ref.
		Soret band	Q bands	
H_2_(TEBOP)^a^	CH_2_Cl_2_	422	517, 554, 593, 651	16
H_2_TTP^b^	CH_2_Cl_2_	420	518, 554, 594, 650	32
H_2_TMPP	CH_2_Cl_2_	423	519, 557, 596, 651	t.w.
H_2_(T_AzP_-HVP)^c^	CH_2_Cl_2_	420	517, 554, 593, 650	33
[Zn(TEBOP)]^a^	CH_2_Cl_2_	424	552 594	16
[Zn(TMPP)]	CH_2_Cl_2_	425	551 592	t.w.
[Zn(T_AzP_-HVP)]^c^	CH_2_Cl_2_	424	551 592	33
[Zn(TPP)(py)]^d^	CH_2_Cl_2_	428	562 602	34
[Zn(TPBP)(DABCO)]^e^^,^^f^	C_6_H_5_Cl	431	564 603	12
[Zn(TPBP)(4,4′-mda)]^e^^,^^g^	CHCl_3_	431	563 604	12
[{Zn(TPBP)}_2_(μ_2_-4,4′-bpy)]^e^	CHCl_3_	430	563 603	12
[{Zn(TPP)}_3_(μ_2_-4,4′-bpy)]^d^	CHCl_3_	425	562 601	15
[Zn(TEBOP)(4,4′-bpy)]^e^	CHCl_3_	430	563 604	16
[Zn(TMPP)(4,4′-bpy)]·CHCl_3_	CH_2_Cl_2_	432	558 604	t.w.

a H_2_(TEBOP) = *meso*-(tetraethyl-4(4-butyryl)oxyphenyl)porphyrin.

b TTP = *meso*-tetra-*p*-tolylporphyrin.

c H_2_(T_Azp_-HVP) = *meso*-tetrakis(3-methoxy-4-((1-phenyl-1*H*-1,2,3-triazol-4-yl)methoxy)phenyl)porphyrin.

d TPP = *meso*-tetraphenylporphyrinate.

e TPBP = *meso*-{tetrakis-[4-(benzoyloxy)phenyl] porphyrin.

f DABCO = 1,4-diazabicyclo[2.2.2]octane.

g 4,4′-mda = 4,4′-diaminodiphenylmethane.

The electronic spectra of H_2_TMPP exhibit a prominent absorption band (B band) at 423 nm known as the Soret band, which corresponds to the allowed transition from the ground state S_o_ to the second excited state S_2_ (S_o_ ← S_2_). Additionally, there are four less intense absorption bands observed at 519, 557, 596, and 651 nm, which are attributed to Qy(1,0), Qy(0,0), Qx(1,0) and Qx(0,0) absorption bands corresponding to the forbidden transitions from the ground state S_o_ to the first excited state S_1_ (S_o_ ← S_1_), respectively. Upon the insertion of the zinc(ii) metal ion into the porphyrin ring, a reduction in both the number and intensity of Q bands was observed, alongside a redshift of up to four nm of the intense Soret band. Thus, the [Zn(TMPP)] obtained complex exhibits electronic spectra with Soret and Q bands at 427 nm, 557 nm and 598 nm, respectively.

For [Zn(TMPP)(4,4′-bpy)]·CHCl_3_ (I), the Soret band displays a noticeable red shift with a *λ*_max_ value of 423 nm which is very close to those of the related Zn(ii)-(4,4′-bpy) *meso*-aryl porphyrin complexes such as [{Zn(TPBP)}_2_(μ_2_-4,4′-bpy)],^12^ [{Zn(TPP)}_3_(μ_2_-4,4′-bpy)],^15^ and [Zn(TPBP)(4,4′-bpy)]^16^ (Table 2). These Soret band values are also very close to those with N-donor neutral axial ligands such as pyridine and DABCO (1,4-diazabicyclo[2.2.2]octane) ligands. This bathochromic shift for the Soret of the later species compared to those of the [Zn(Porph)] starting materials may be attributed to an increase in the π-conjugation resulting from the addition of the N-donor axial ligand.

The optical gap energy (*E*_g-opt_) values of H_2_TMPP, [Zn(TMPP)] and [Zn^II^(TMPP)(4,4′-bpy)]·CHCl_3_ (I) calculated using the Tauc method^35^ are 1.848, 1.946 and 1.957 eV, respectively (Fig. S9†). These values indicate that our three porphyrinic compounds are considered semi-conductors, which is typical for all porphyrins and metalloporphyrins.

### 3.4 Fluorescence spectroscopy

**Fig. 2:**
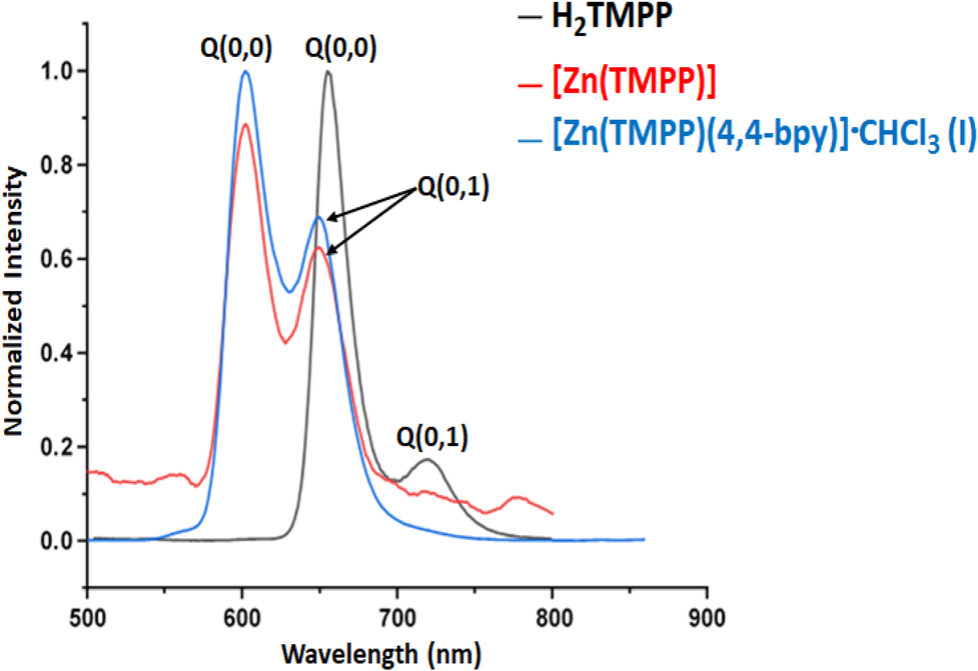
The fluorescence spectra of H_2_TMPP, [Zn(TMPP)] and [Zn(TMPP)(4,4′-bpy)]·CHCl_3_ (I) in CH_2_Cl_2_ (*ca.* 10^−6^ M).

The fluorescence emission spectrum of free base porphyrin H_2_TMPP recorded in dichloromethane solvent at room temperature upon photoexcitation at 420 nm exhibits two emission bands Q(0,0) and Q(0,1) corresponding to the S_1_ ← S_o_ transition (Fig. 2). These emission bands exhibit maximum emission wavelengths values of 655 nm and 721 nm corresponding to Q(0,0) and Q(0,1), respectively. The metalloporphyrin [Zn(TMPP)] gave two split emission bands Q(0,0) and Q(0,1) with values of 602 and 650 nm, respectively. Axially ligated metalloporphyrin (complex I) give rise to two emission from the S_2_ excited state to the fundamental state S_0_, with one centered at 601 nm (resulting from S_2_ [Q(0,0)] ← S_0_) and the other at 649 nm (stemming from S_2_ [Q(0,1)] ← S_0_) when excited at 430 nm for [Zn(TMPP)(4,4′-bpy)]·CHCl_3_ (complex I). The fluorescence quantum yields (*Φ*_f_) for H_2_TMPP, [Zn(TMPP)] and [Zn(TMPP)(4,4′-bpy)]·CHCl_3_ are 0.082, 0.033 and 0.028, respectively, with lifetimes of 7.15 ns for H_2_TMPP, 1.5 ns for [Zn(TMPP)] and 1.3 ns for complex I (Table 3). These values fall within the typical range for *meso*-arylporphyrins and their Zn(ii) complexes.

**Table 3:** Emission data for H_2_TMPP, [Zn(TMPP)], [Zn(TMPP)(4,4′-bpy)]·CHCl_3_ (I) and a selection of *meso*-tetraarylporphyrins compounds

Compound	*λ*_max_ (nm)		*Φ*_f_	*τ*_f_ (ns)	Ref.
	Q(0,0)	Q(0,1)			
***meso*-Arylporphyrins**					
H_2_TPP^a^	653	722	0.12	9.60	36
H_2_TClPP^b^	652	714	0.089	7.42	37
H_2_TTP^c^	657	721	0.098	7.90	37
H_2_TMPP	655	721	0.082	7.15	t.w.

**Zinc(** **ii** **) *meso*-arylporphyrins**					
[Zn(TMPP)]	602	650	0.033	1.5	t.w.
[Zn(TTP)]^c^	600	648	0.030	1.6	37
[Zn(TPBP)]^d^	606	654	0.027	1.6	12
[Zn(TPBP)(DABCO)]^d^^,^^e^	612	660	0.039	1.3	12
[Zn(TPBP)(pyz)_2_]^d^^,^^f^	596	644	0.049	1.6	12
[Zn(TPBP)(4-CNpy)]^d^^,^^g^	596	645	0.041	1.5	12
[{Zn(TPBP)}_2_(μ_2_-4.4-bpy)]^d^	596	645	0.044	1.5	12
[Zn(TEBOP)(4,4′-bpy)]^i^	603	651	0.028	1.2	16
[Zn(TMPP)(4,4′-bpy)]·CHCl_3_ (I)	601	649	0.035	1.3	t.w.

a H_2_TPP = *meso*-tetraphenylporpyrin.

b H_2_TClPP = *meso*-tetra(4-chlorophenyl)porphyrin.

c H_2_TTP = *meso*-tetra(*p*-tolyl)porphyrin.

d TPBP = *meso*-tetrakis(4-*tert*-butylphenyl)porphyrinate.

e DABCO = 1,4-diazabicyclo[2.2.2]octane.

f pyz = pyrazine.

g 4-CNpy = 4-cyanopyridine.

i TEBOP = *meso*-(tetraethyl-4(4-butyryl)oxyphenyl)porphyrin.

### 3.5 Cyclic voltammetry of complex I

The cyclic voltammetry spectrum of complex I is depicted in Fig. S10† while in Table 4 is given the half-potential (*E*_1/2_) values of the oxidation and reduction waves of complex I and several zinc(ii) porphyrin complexes.

**Table 4:** Electrochemical data^a^ for [Zn(TMPP)(4,4′-bpy)]·CHCl_3_ (I) and a selection of Zn(ii) *meso*-metalloporphyrins

Complex	Oxidations 1st porph oxid	2nd porph oxid	Reductions 3rd porph oxid	1st porph red		Ref. 2nd porph red
	(O1,R1)	(O2,R2)	(R3,O3)	(R4,O4)	(R5,O5)	
	*E*_1/2_ b	*E*_1/2_	*E*_1/2_	*E*_1/2_	*E*_1/2_	
[Zn(TPP)(HIm)]^c^^,^^d^	0.65	1.35	—	−1.34*	−1.67*	34
[Zn(TMP)(2-MeIm)]^e^^,^^f^	0.58*	1.18*	—	−1.55*	—	34
[Zn(TPP)(CN)]^−^c	0.65	1.06	1.38	−1.51	−1.77	37
[Zn(TPBP)(DABCO)]^g^^,^^i^	0.84	1.12	—	−1.33*	—	12
[Zn(TPBP)(pyz)_2_]^g^^,^^j^	0.82	1.12	1.38*	−1.34*	—	12
[Zn(TPBP)(4,4′-mda)]^g^^,^^k^	0.81	1.28*	—	−1.31*	−1.69	12
[Zn(TPBP)(4-CNpy)]^g^^,^^l^	0.81	1.10	1.36	−1.52	−1.74	12
[{Zn(TPBP)}_2_(μ_2_-4,4′-bpy)]^g^	0.81	1.13	1.38*	−1.30*	—	12
[Zn(TMPP)(4,4′-bpy)] (I)	0.82	0.98	1.39	−1.38	—	This work

a Potentials are reported *versus* SCE.

b *E*_1/2_ = half wave potential.

c TPP = *meso*-tetraphenylporphyrinate.

d HIm = imidazole.

e TMP = *meso*-tetramesitylporphyrin.

f 2-MeIm = 2-methylimidazole.

g TPBP = *meso*-tetrakis(4-*tert*-butylphenyl)porphyrinate.

i DABCO = 1,4-diazabicyclo[2.2.2]octane.

j pyz = pyrazine.

k 4,4′-mda = 4,4′-diaminodiphenylmethane.

l 4-CNpy = 4-cyanopyridine, *: irreversible wave.

Zinc(ii) metalloporphyrins exhibit two or three one-electron reversible or quasi-reversible oxidation waves and one or two reversible or quasi-reversible reduction waves corresponding to the oxidation and the reduction of the porphyrin macrocycle.^34^ For our Zn(ii)-4,4′-bpy-TMPP derivative, the *E*_1/2_ values of the first, second and third oxidation waves are 0.82, 0.98 and 1.39 V which are very close to the related Zn(ii) metalloporphyrins reported in Table 4. In the anodic region of the voltammogram of complex I, the quasi-reversible wave with *E*_1/2_ value of −1.38 V is attributed to the first reduction of the porphyrin core of the TMPP porphyrinate. This value is also in the range observed for pentacoordinated and hexacoordinated zinc(ii) porphyrin coordination compounds. Notably, the UV-Vis data and those of the cyclic voltammetry of zinc(ii) metalloporphyrins are very close regardless the nature of the *meso*-arylporphyrins and the axial ligands.

### 3.6 X-ray structures of [Zn(TMPP)(4,4′-bpy)]·CHCl_3_ (I)

**Fig. 3:**
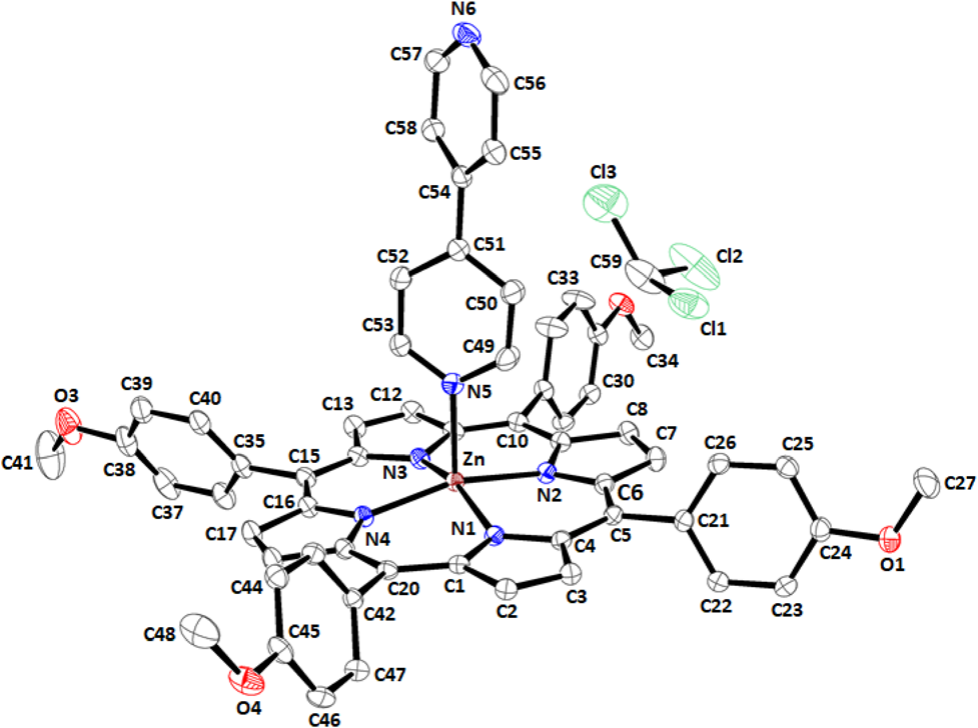
ORTEP drawing of [Zn(TMPP)(4,4′-bpy)]·CHCl_3_ (I) with thermal ellipsoids drawn at 40% probability.

Complex I crystallizes in the monoclinic space group with the centrosymmetric *P*2_1_/*c* space group. The number of formula units by cell is *Z* = 4, and the asymmetric unit is made by one [Zn(TMPP)(4,4′-bpy)] molecule and one chloroform solvent molecule leading to the formula [Zn(TMPP)(4,4′-bpy)]·CHCl_3_ (I). An Ortep view of I is depicted in Fig. 3 while a selection of distances and angles of complex I is given in Table S3.†

The hydrogen atoms are removed for clarity.

As mentioned in the introduction, 4,4′-bpy zinc(ii) metalloporphyrins exhibit four types of solid-state molecular structures which are: monomers, dimers, trimmers and polymers. The majority of these 4,4′-bpy zinc(ii) porphyrin complexes are dimers type [{Zn(Porph)}_2_(μ_2_-4,4′-bpy)], *e.g.* [{Zn(TPP)}_2_(μ_2_-4,4′-bpy)],^17^ only one trimer complex is reported which is [{Zn(TPP)}_3_(μ_2_-4,4′-bpy)]^15^ and one polymer type is known which is {[Zn(TMP)(4,4′-bpy)]}_*n*_ (TMP = *meso*-tetrakis(2,4,6-trimethylphenyl)porphyrin).^32^ For monomers type [Zn(Porph)(4,4′-bpy)], besides our [Zn(TMPP)(4,4′-byp)] (I) species, only one example is reported in the literature which is [Zn(TEBOP)(4,4′-bpy)] (TEBOP = *meso*-(tetraethyl-4(4-butyryl)oxyphenyl)porphyrin).^16^ It is clear from these examples that Zn(ii)-(4,4′-bpy)-Porph complexes can adopt either monomer, dimer, trimer or polymer structure types. The prediction of the types of these Zn(ii)-(4,4′-bpy) metalloporphyrins based on the nature of the substituents on the para-positions of a *meso*-arylporphyrin or on the β-pyrrolic positions of a porphyrin is not possible. Kinetic and theoretical investigations are needed to understand this phenomenon.

The average equatorial distance between the zinc(ii) central ion and the nitrogen atoms of the porphyrin ring (Zn^__^Np) of complex I is 2.0682(17) Å which is very close to those of the related hexacoordinated and pentacoordinated 4,4′-bipyrine zinc(ii) metalloporphyrins (Table S3†). The Zn^__^N(4,4′-bpy) distance value of 2.1441(17) Å for complex I is very close to that of the related zinc(ii) porphyrin complexes reported in Table 5. The reported dihedral angle φ (Figure S11†) values between the two pyridyl groups of the 4,4′-bpy axial ligand range between 0 and 38°, while for complex I, the φ angle is 41.78(4)° which is slightly higher. Nevertheless, for the magnesium(ii) 4,4′-bipyrine complex {[Mg(TPBP)(4,4′-bpy)_2_]}_*n*_ the *φ* value is quite higher than that of complex I with a value of 59.60°.^42^

**Table 5:** Selected bond lengths [Å] and angles [°] for [Zn(TMPP)(4,4′-bpy)]·CHCl_3_ (I) and several related 4,4′-bipyridine *meso*-arylporphyrin complexes

Complex	M^__^N_p_^a^	M^__^N^b^	M^__^P_C_^c^	*φ* d(°)	Ref.
**(4,4′-Bipyridine) zinc(** **ii** **) metalloporphyrins**					
[Zn(TEBOP)(4,4(-bpy)]^e^	2.0675(3)	2.151(2)	0.29	38)	16
[Zn(TMPP)(4,4′-bpy)]] (I)	2.0682(17)	2.1441(17)	0.3083(4)	41.78(4)	This work
[{Zn(TPBP)}_2_(μ_2_-4,4′-bpy)]^f^	2.063(6)	2.178(6)	0.329(2)	0	12
[{Zn(TPP)}_2_(μ_2_-4,4′-bpy)]^g^	2.081	2.169	0.333	37.84	17
[{Zn(T(OH)PP}_2_(μ_2_-4,4′-bpy)]^i^	2.047/2.041	2.134/2.144	0.306/0.308	0.0	38
[{Zn(TPP)}_3_(μ_2_-4,4′-bpy)]^g^	2.036/2.054	2.185/2.490	0.319/0.003	23.61/23.65	15
	2.050	2.185	0.317		
{[Zn(TMP)(4,4′-bpy)]}_*n*_^j^	2.059	2.371	0.0	0.0	35

**(4,4′-Bipyridine) metalloporphyrins**					
[Co^III^(TpivPP)Cl(4,4′-bpy)]^k^	1.983	2.028	—	29.78	39
{[Co^II^(TPP)(4,4′-bpy)]}_*n*_^g^	1.993	2.342	—	37.72	40
{[Fe(TPP)(4,4′-bpy)]}_*n*_^g^	1.990	1.985	—	29.44	41
{[Mg(TPBP)(4,4′-bpy)_2_]}_*n*_^f^	2.065	2.319/2.290	—	59.60	42
[Ni(TCPP)(4,4′-bpy)_2_]^l^	2.050	2.197	—	24.67	43

a M^__^Np = average equatorial M–N pyrrole bond length.

b M^__^N_L_ = distance between the metal atom and the nitrogen atom of axial ligand.

c M^__^P_C_ = distance between Mg and the mean plane made by the 24-atom core of the porphyrin (P_C_).

d φ = the diedral angle between the two pyridyl groups of the 4,4′-bpy.

e TEBOP = *meso*-(tetraethyl-4(4-butyryl)oxyphenyl)porphyrin.

f TPBP = *meso*-tetrakis(4-*tert*-butylphenyl)porphyrinate.

g TPP = *meso*-tetraphenylporphyrinate.

i T(OH)PP = *meso*-tetrakis(4-hydroxyphenyl)porphyrinate.

j TMP = *meso*-tetramesitylporphyrinate.

k TpivPP = α,α,α,α-tetrakis(*o*-pivalamidophenyl)porphinate.

l TCPP = 5,10,15,20-tetrakis(4-carboxyphenyl)porphyrinato.

**Fig. 4:**
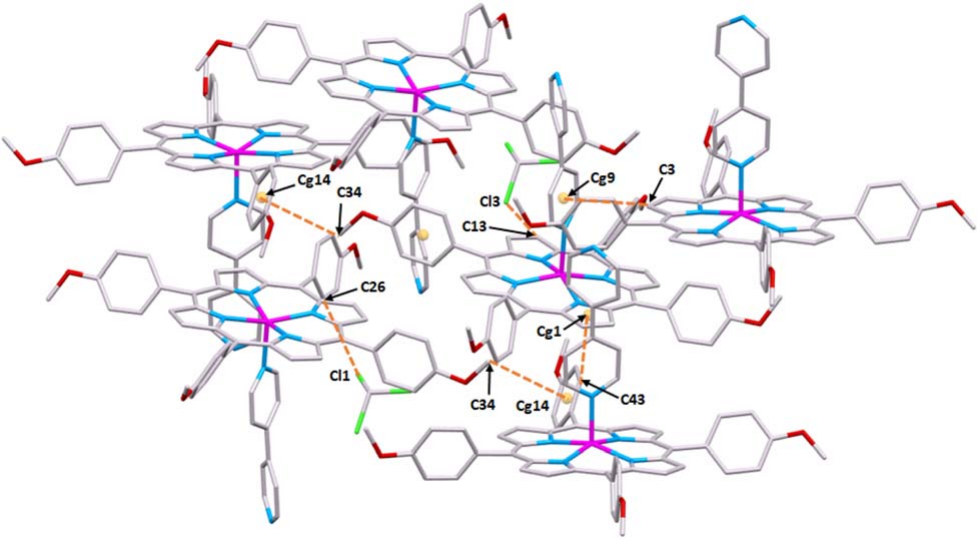
Packing diagram of complex I showing the intermolecular interactions types C–H⋯Cl and C–H⋯Cg (Cg = centroid of phenyl groups of the TMPP porphyrinate) linking differents [Zn(TMPP)(4,4′-bpy)] and CHCl_3_ molecules.

Fig. S12† illustrates the crystal packing of complex I along the *b* direction which is stabilized by intermolecular interactions types C^__^H⋯Cl, C^__^H⋯O, C^__^H⋯N and C^__^H⋯Cg (Cg are the centroids of a pyrrole ring, a phenyl ring or a pyridyl ring) including the [Zn(TMPP)(4,4′-bpy)] complexes and the chloroform solvent molecules (Fig. 4 and Table S4†).

### 3.7 Hirshfeld surface analysis

The Hirshfeld Surface (HS) and 2D fingerprint plots of complex I was investigated using The Crystal Explorer 17.5 program.^44^Eqn (1) gives the normalized contact distance *d*_norm_ as a function of the nearest atom outside (*d*_e_), the inside (*d*_i_) the Hirshfeld surface and the van der Waals radii (*r*^vdW^):1
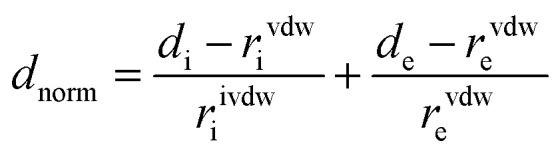


**Fig. 5:**
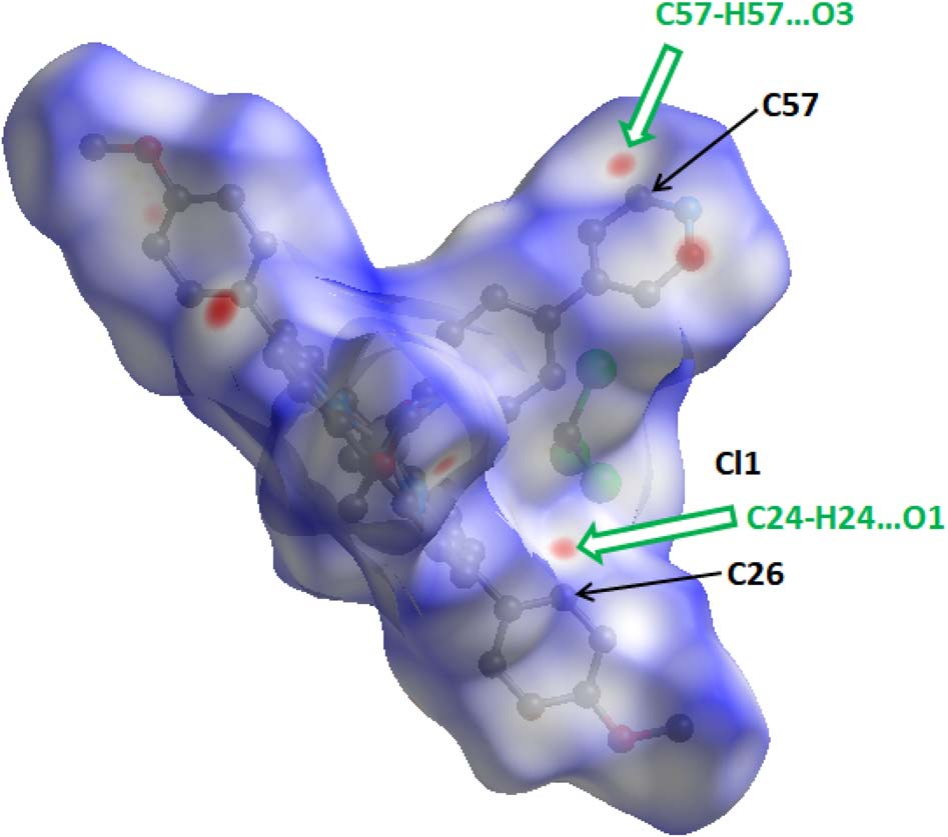
Representation of the Hirshfeld surface for complex I plotted over *d*_norm_ in the range −0.1949 to 1.5547 a.u.

The shorter contacts than the sum of the van der Waals radii are shown in red in the Hirshfeld surface, while the longer and closer to the van der Waals contacts are indicated in blue and white colors (Fig. 5).

We notice that by using the Crystal Explorer 17.5 program or the PLATON program (see the X-ray molecular structure section) we got practically the same types of the intermolecular interactions. In Fig. S13† are depicted the two-dimensional fingerprint plots for complex I showing that the major types of intermolecular contacts responsible of the stability of the crystal lattice of I are: H⋯H (45.8%), H⋯C (25.5%), H⋯Cl (8.7%) H⋯O (7.2%) and H⋯N (6.4%).

The sharp index mapped on HS of complex I (Fig. S14-a†) show the absence blue and red triangles indicating that there are no π⋯π stacking interactions in the crystal lattice. The absence of flat surfaces patches in the curvedness plot (Fig. S14-b†) is an indication of the absence of planar stacking.

The absence of planar stacking is confirmed by the fact that there are no flat surfaces patches in the curvedness plot (Fig. S14-b†).

### 3.8 Photocatalytic degradation of methylene blue

**Fig. 6:**
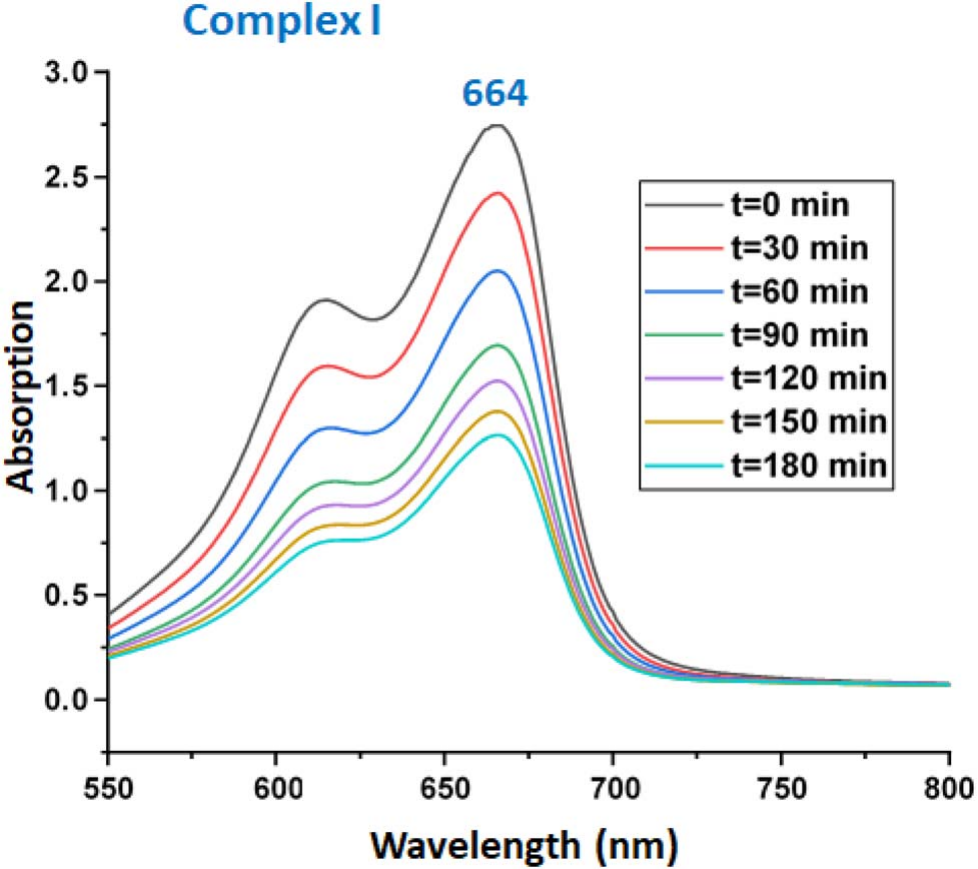
Change in absorbance intensity of the MB dye at *λ*_max_ (664 nm) under visible light in the presence of compound I (5 mg). The MB concentration was 20 mg l^−1^ with a pH of 6.

We investigated the photocatalytic degradation of the MB dye using our Zn(ii)-TMPP-4,4′-bpy species (I) as photocatalyst at room temperature, pH = 6 and a pure blue led lamp irradiation (emitting monochromatic light, *λ* = 450 nm). The masse of complex I used is 5 mg (0.0052 mmol), the initial dye concentration is 20 mg L^−1^. As shown by Fig. 6, the MB concentration started decreasing after 30 min of reaction indicating that a small quantity of the dye is adsorbed by complex I.

**Fig. 7:**
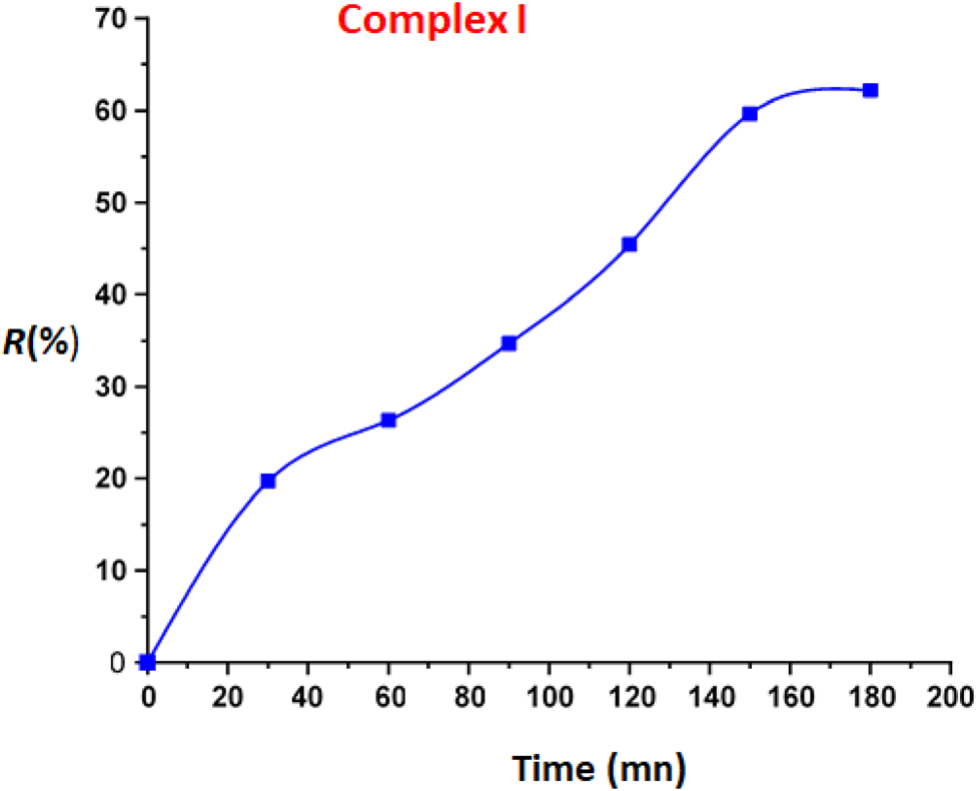
Variation of the photodegradation yield (%*R*) of the MB dye under visible light in the presence of compound I as function of time.

The UV-Vis spectroscopy was used to monitored the photodegradation of the MB dye using complex I as photocatalyst and especially the variation of the absorption at *λ*_max_ equal to 664 nm (corresponding to the highest absorption band of the MB organic species) in function of time. After 180 min of reaction, the decomposition efficiency for the MB dye reaches 63% (Fig. 7).

The degradation yield (*R*%) is calculated using the eqn (2) provided below.2
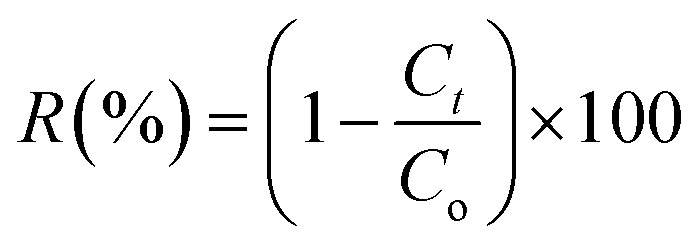
where *C*_o_ and *C*_t_ are the concentrations of the MB dye at the instant *t* = 0 and the instant = *t*, respectively.

The kinetic investigation shows that degradation reaction of the MB dye reaction fellows the pseudo-second order kinetic model and the *k* was calculated using the following Langmuir–Hinshelwood eqn (3):3
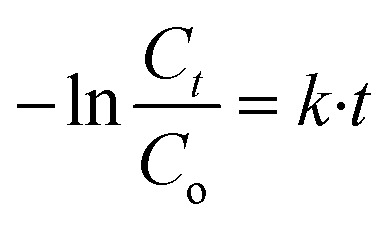
where *C*_o_ and *C*_t_ are the concentrations of the methylene blue (MB) at times *t* min and *t* = 0 min.

**Fig. 8:**
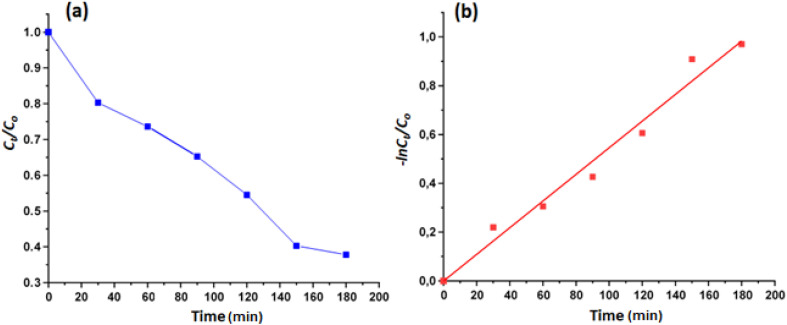
(a): Evolution of *C*_*t*_/*C*_o_ as a function of time, (b): variation of −ln(*C*_*t*_/*C*_o_) as function of time.

The calculated rate constant *k* value is 0.00546 min^−1^ with *R*^2^ = 0.9760 (Fig. 8).

The reusability of [Zn(TMPP)(4,4′-bpy)]·CHCl_3_ (I) as a photocatalyst for the degradation of methylene blue (MB) dye was assessed. After each catalytic cycle, complex I was recovered *via* filtration, thoroughly rinsed with distilled water, and dried in an oven at 60 °C. The photocatalyst demonstrated consistent performance over three successive cycles under identical conditions (Fig. S15†).

The mechanism of heterogeneous photocatalysis, illustrated in Fig. S16,† involves the activation of complex I through the absorption of light energy with a wavelength equal to or greater than the material's band gap energy (*hν* ≥ *E*_g_). Upon activation, an electron–hole pair (e^−^/h^+^) is generated *via* the excitation of an electron from the valence band to the conduction band. The excited electron reacts with oxygen molecules adsorbed on the surface of the porphyrinic complex, while the hole (h^+^) interacts with surface hydroxyl ions (OH^−^), producing highly reactive hydroxyl radicals (OH˙). These radicals are primarily responsible for degrading the MB dye. The generation of (OH˙) as reactive oxygen species (ROS) is also facilitated by the efficient electron transfer in the Zn–porphyrin complex, which enhances their photocatalytic activity. The reaction between hydroxyl radicals and the MB dye results in the formation of carbon dioxide (CO_2_) and water (H_2_O), the main mineralization products.

### 3.9 Catalytic oxidative degradation of MB dye

#### 3.9.1. Effect of the initial MB dye concentration

**Fig. 9:**
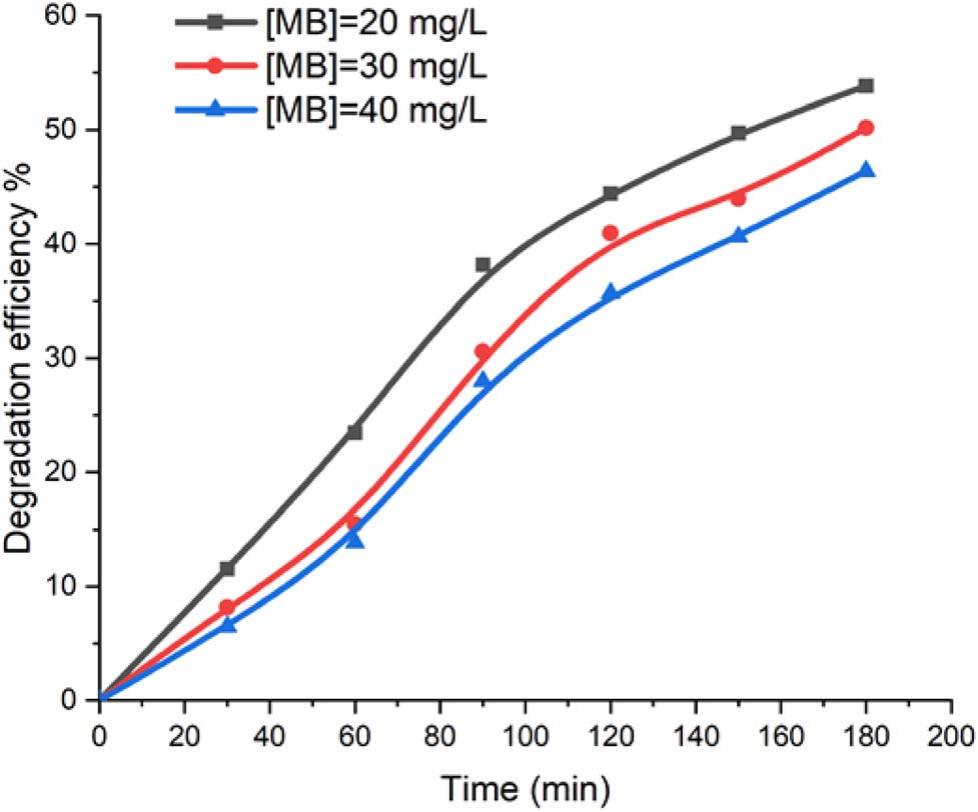
Variation in color removal as a function of the initial dye concentration was examined under the following conditions: hydrogen peroxide concentration (*C*_0_) of 4 mL L^−1^, catalyst mass of 5 mg, and pH 6.

During the degradation of methylene blue (MB) dye, the reaction involves zinc(ii) metalloporphyrin (I) (5 mg, 0.0052 mmol) in the presence of an aqueous solution of H_2_O_2_ (4 mL L^−1^) at pH 6. Fig. 9 illustrates the relationship between the color removal and the initial MB dye concentration. The results indicate that as the initial dye concentration increases, the degradation kinetics of MB slow down. This behavior can be attributed to the higher number of MB molecules at elevated concentrations, while the availability of hydroxyl radicals remains constant. Consequently, both the reaction rate and the efficiency of dye discoloration decline with increasing MB concentration.

#### 3.9.2. Effects of pH

To investigate the effect of pH on the degradation of the MB dye by complex I, aqueous solutions were prepared with pH values ranging from 2 to 10, adjusted using 0.1 M of hydrochloric acid (HCl) and 0.1 of sodium hydroxide (NaOH). Five solutions with initially determined pH values (2, 4, 6, 8 and 10), without any subsequent changes or pH control during the process, were used to study the effect of pH on the dye oxidation. Fig. S17† presents the dye removal efficiency at varying initial pH levels of the solution over different reaction times. The results reveal that the highest dye conversion was achieved at pH 6, with maximum removal occurring 180 minutes after the reaction commenced.

#### 3.9.3. Effects of temperature

We investigated the impact of temperature on the degradation of MB dye in presence of complex I. The temperatures employed were 25, 35 and 45 °C, as shown in Fig. S18.† As predicted, raising the temperature from 25 °C to 45 °C led to an improvement of the degradation efficiency of MB dye, with an increase from 53.84% to 57.98%. This is because higher temperatures increase the rate of reaction between hydrogen peroxide H_2_O_2_ and the zinc metal center. This in turn augments the rate of generation of the hydroxyl radical oxidizing species.

The Langmuir–Hinshelwood is given by the following eqn (4):4
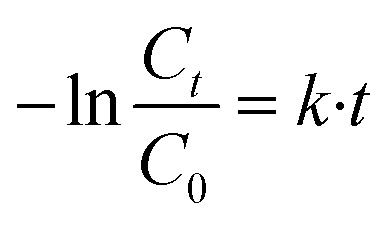


**Fig. 10:**
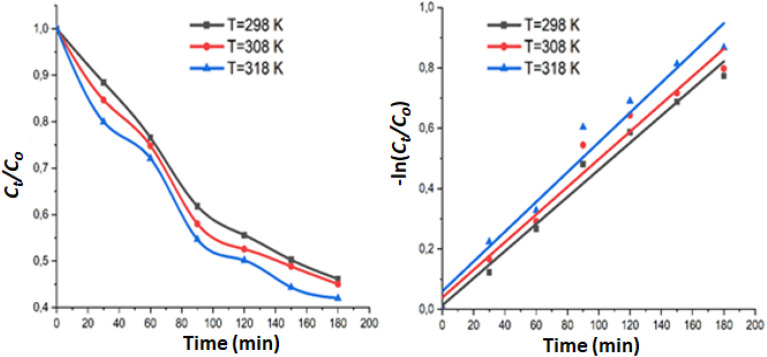
Variation of *C*_*t*_/*C*_o_ and −ln(*C*_*t*_/*C*_o_) as a function of time at various temperatures. The lines represent the fitted data using the Pseudo-Second Order kinetic model.

The experimental data were analyzed using this formula at various reaction temperatures. Fig. 10 depicts the plots of *C*_t_/*C*_o_ and −ln(*C*_t_/*C*_o_) as functions of time, both demonstrating high correlation coefficients. These findings indicate that the degradation reaction adheres to a pseudo-first-order kinetic model. The apparent rate *k* constants were derived from the slopes of the linear regression curves at 298 K, 308 K, and 318 K. The calculated *k* values for the degradation of MB dye were 0.00449 min^−1^ (*R*^2^ = 0.9832), 0.00458 min^−1^ (*R*^2^ = 0.9667) and 0.00493 min^−1^ (*R*^2^ = 0.9639) at 298, 308 and 318 K, respectively.

#### 3.9.4. Degradation with optimal conditions

**Fig. 11:**
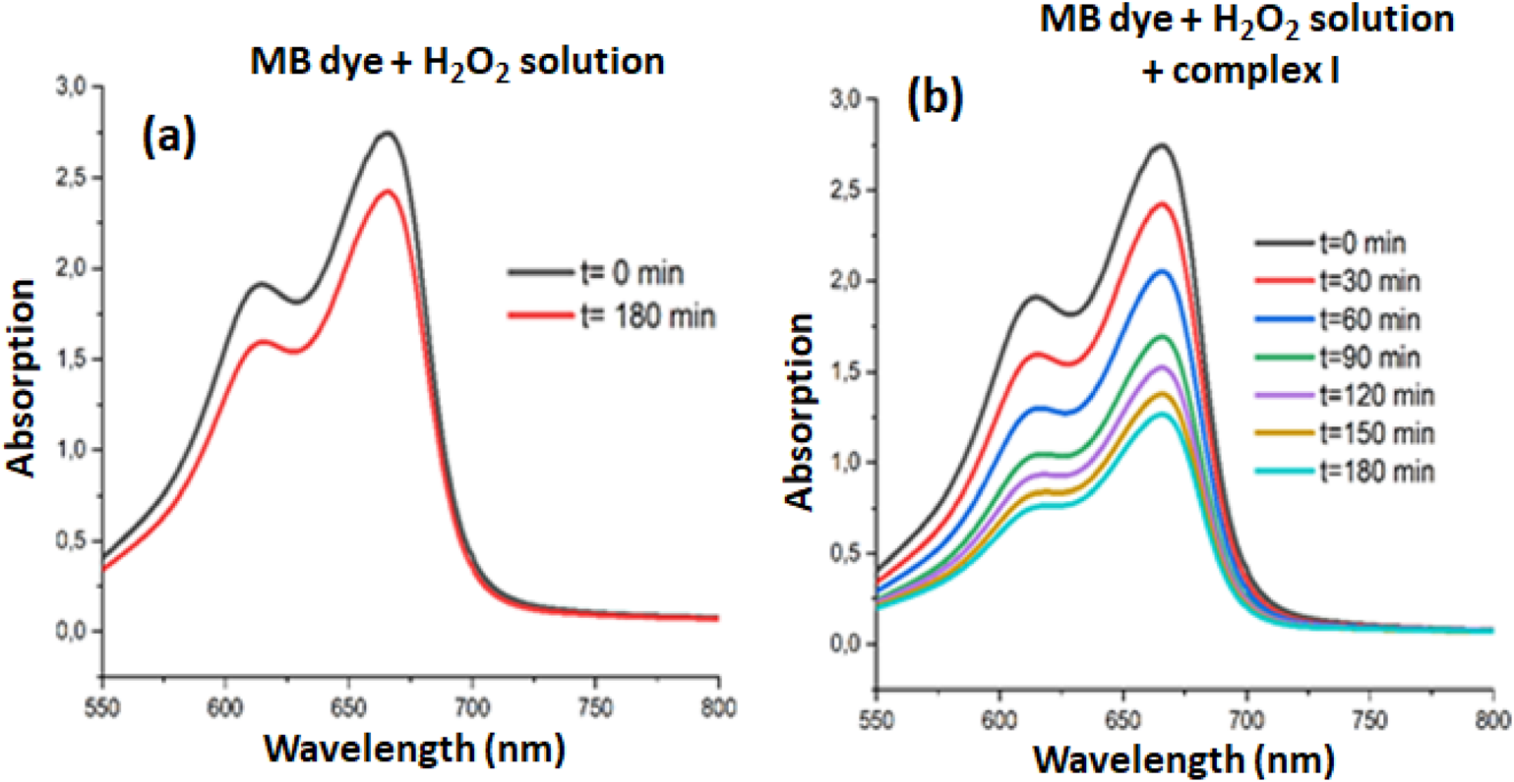
Variation of the intensity of *λ*_max_ (664 nm) during the catalytic degradation of the MB dye in the presence of H_2_O_2_ aqueous solution and the absence of complex I (a) and the presence of complex I (b).

The degradation study of the MB dye was carried out at room temperature using an aqueous H_2_O_2_ solution under optimal conditions which are: 5 mg of complex I (0.0052 mmol) and 10 mL of an aqueous solution of MB dye with a concentration of 20 mg L^−1^, the hydrogen peroxide aqueous solution concentration utilized was 4 mL L^−1^, and the pH was set at 6. Fig. 11-a illustrates the absorption curves of the MB dye in the presence of H_2_O_2_ without a catalyst (blank experiment) over 180 min while Fig. 11-b represents the absorption curves of the MB dye in the presence of H_2_O_2_ and complex I for various degradation times. It is evident from these figures that the absorption remains essentially unchanged for a duration of 180 minutes without complex I used as catalyst. When our Zn(ii)-4,4′-bpy-TMPP complex is used, the obtained degradation yield is 53.84% after 180 minutes.

#### 3.9.5. Comparison of our procedure with other reported methods

Notably, the important number of investigations concerning the MB degradation.^45–49^Table 6 shows the optimal reaction conditions, degradation yield, and reaction time for several degradation systems for the MB dye.

**Table 6:** Examples of degradation systems employed for the MB dye degradation with the optimal reaction conditions and yields

Degradation system	Degradation method	Degradation yield, time reaction	Ref.
System used: MnTCPPOAc@ MWCNT	Catalytic oxidation by H_2_O_2_	98% (720 min)	45
System used: FeTCPPCl@MWCNT	Catalytic oxidation by H_2_O_2_	30% (720 min)	45
System used: [Co^II^(TMAPP)]	Catalytic oxidation by H_2_O_2_	44% (240 min)	46
System used: BiFeO_3_	Photodegrdation	70.8% (360 min)	47
System used: BiFeO_3_/GdFeO_3_	Photodegrdation	52% (360 min)	48
System used: [Ni^II^(TAMPP)]	Catalytic oxidation by H_2_O_2_	73% (90 min)	49
System used: [Zn^II^(TMPP)(4,4′-bpy)] (I) (H_2_O_2_ solution)	Photodegradation	63% (180 min)	t.w.
System used: [ZnII(TMPP)(4,4′-bpy)]·CHCl_3_ (I)	Catalytic oxidation by H_2_O_2_	53.84% (180 min)	t.w.

The catalytic degradation of MB dye, using [Zn(TMPP)(4,4′-bpy)]·CHCl_3_ (I) along with an aqueous hydrogen peroxide solution, yields approximately 54%. While this yield is deemed acceptable in comparison to other H_2_O_2_ oxidation methods for this dye as listed in Table 6, it is understandably lower than the yields achieved by photocatalytic systems, as expected.

#### 3.9.6. Activation energy and thermodynamic variables

For a more comprehensive understanding of the degradation phenomenon, we used the pseudo-first-order model to calculate the kinetic parameters. The activation energy (*E*_a_) was determined by applying the Arrhenius law (eqn (5)). Eyring's equation (eqn (6) and (7)) was employed to calculate the thermodynamic activation parameters, including enthalpy (ΔH*), entropy (ΔS*), and Gibbs free energy (ΔG*).5
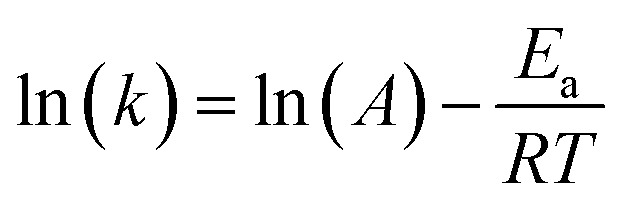
6
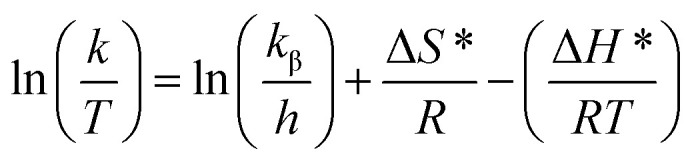

7 ΔG* = Δ*H* − TΔS*

Here, h and *k*_β_ denoting Planck's and Boltzmann's constants, respectively, *k* is the kinetic rate, *R* is the gas constant and *A* is the Arrhenius constant. In Table 7 are summarized the obtained thermodynamic parameters and activation energy values. It was found that the free energy is positive at 298, 308 and 318 K indicating that the degradation of MB dye with H_2_O_2_ is not a spontaneous reaction. The free enthalpy values are positive is an indication that the decolonization process is endothermic which increases with the temperature. As a consequence of the negative value of the entropy, during the degradation process, there is a reduction of the disorder. The value for activation energy is 3682.74 J mol^−1^.

**Table 7:** Thermodynamic parameters (ΔS*, ΔH*, ΔG*) and activation energy (*E*_a_) for complex I

Temperature (K)	298	308	318
*k* (min^−1^)	000 449	000 458	000 493
*E*_a_ (J mol^−1^)	368 274	368 274	368 274
ΔS* (J mol^−1^ K^−1^)	−28 608	−28 608	−28 608
ΔH* (J mol^−1^)	112 383	112 383	112 383
ΔG* (J mol^−1^)	8 637 567	8 923 647	9 209 727

### 3.10 Electrochemical sensor application of [Zn(TMPP)(4,4′-bpy)]·CHCl_3_ (I)

#### 3.10.1 Characterization of the modified glassy carbon electrode

**Fig. 12:**
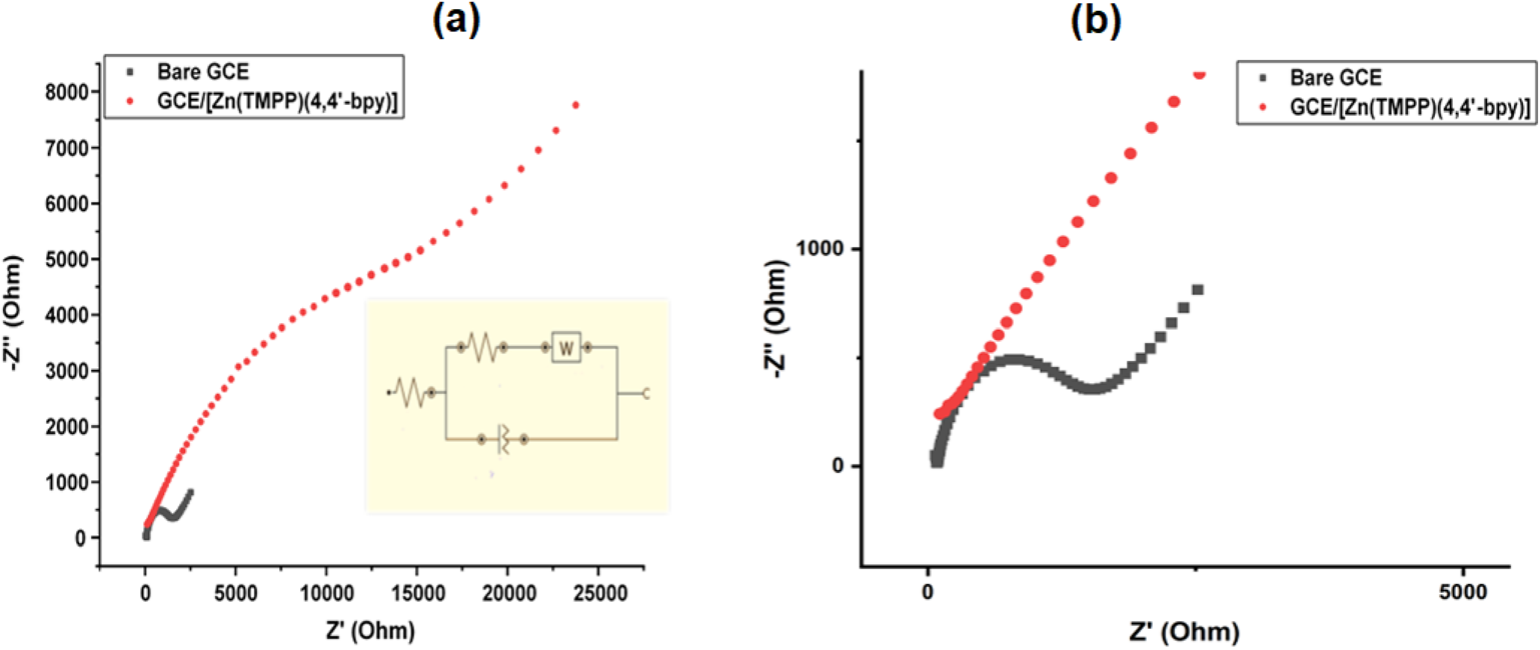
(a) Nyquist plots were obtained for a 5 mM [Fe(CN)_6_]^3−/4^ solution in 0.1 M of KCl of bare GCE and GCE/[Zn(TMPP)(4,4′-bpy)]·CHCl_3_. Measurements were conducted over a frequency range of 0.05 to 75 000 Hz. The inset illustrates the equivalent circuit model [(R(Q[RW]] used for data analysis. (b) Impedance graph with inset (0–5000 ohms).

Cyclic voltammetry (CV) and electrochemical impedance spectroscopy (EIS) were employed to investigate the behavior of various modified electrodes. Both techniques were performed in a 0.1 M KCl solution containing 5 mM [Fe(CN)_6_]^3−/4−^. As illustrated in Fig. S19,† the bare glassy carbon electrode (GCE) displayed clearly defined redox peaks at 0.281 V and 0.167 V, typical of a quasi-reversible CV. After deposition of the [Zn(TMPP)(4,4′-bpy)]·CHCl_3_ (I) film, the electrochemical signals corresponding to the [Fe(CN)_6_]^3−/4−^ redox couple on the modified surface were no longer visible, indicating successful preparation of the [Zn(TMPP)(4,4′-bpy)]·CHCl_3_ film. In Fig. 12, impedance spectra were analyzed using equivalent circuits with the NOVA 2.1 software,^50^ and the results are summarized in Table 8. The equivalent circuit model provided estimates for various parameters, including solution resistance (*R*_s_), charge transfer resistance (*R*_tc_), constant phase element (CPE), and diffusion impedance (*W*). Nyquist plots for the bare GCE show a straight line at low frequencies and a small semicircle at higher frequencies. Upon modification with the deposited layer, the semicircle in the high-frequency region increases, which reflects the impact of the film and confirms its excellent electrical conductivity.

**Table 8:** Electrochemical parameters of bare GCE and GCE/[Zn(TMPP)(4,4′-bpy)]·CHCl_3_ obtained from the analysis of impedance data with the equivalent circuit

Electrodes	*R*_S_ (Ω)	CPE (μF)	*R*_tc_ (kΩ)	*W*(μF)
Bare GCE	81.841	13.15	5.04	325
GCE/[Zn(TMPP)(4,4′-bpy)]·CHCl_3_	−150.8	12.76	28.7	222

#### 3.10.2. Electrochemical sensing of dopamine (DA) at GCE/[Zn(TMPP)(4,4′-bpy)]·CHCl_3_

**Fig. 13:**
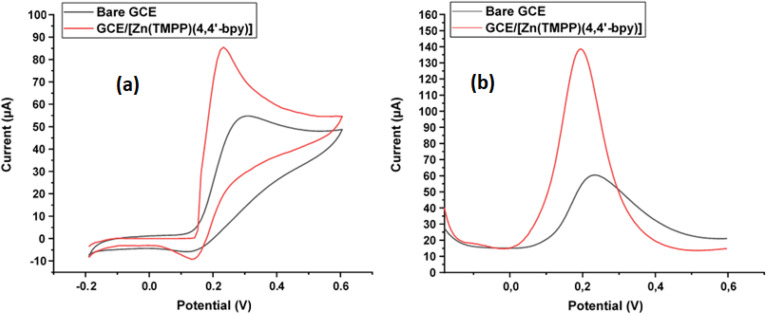
(a): CV and (b): SWV spectra performed on GCE electrodes with 1 mM dopamine in a PBS (0.1 mol L^−1^, pH = 7) solution.

It is noteworthy that Dopamine sensing is crucial due to its significant role as a neurotransmitter in the human central nervous system. Abnormal levels of dopamine can lead to various medical and behavioral issues, making its quantification essential. Dopamine is involved in functions like motor control, reward, and reinforcement and its abnormal levels are linked to neurological diseases such as Parkinson's, schizophrenia, Alzheimer's, stress, and depression.^51^ Electrochemical biosensing is a valuable method for dopamine detection, offering robustness, selectivity, sensitivity, and real-time measurements. Sensors for dopamine need to be sensitive, selective, and capable of discriminating dopamine from interfering substances like ascorbic acid or uric acid.^52^ The electrochemical responses of bare GCE and GCE/[Zn(TMPP)(4,4′-bpy)]·CHCl_3_ towards dopamine recognition were investigated using cyclic voltammetry (CV) in 0.1 M PBS with a pH of 7, after a 5 minutes incubation time. The CV responses of dopamine at bare electrode and GCE/[Zn(TMPP)(4,4′-bpy)]·CHCl_3_ are shown in Fig. 13. It is evident that the GCE/[Zn(TMPP)(4,4′-bpy)]·CHCl_3_ modified electrode exhibits a higher oxidation peak current than the bare electrode. This increase in peak current upon modification with [Zn(TMPP)(4,4′-bpy)]·CHCl_3_ can be attributed to the enhanced electrical conductivity of the deposited film. Furthermore, square wave voltammetry (SWV) measurements revealed that the modified electrode demonstrates superior recognition performance for dopamine, as shown in Fig. 13. The observed negative shift in oxidation potential suggests that dopamine oxidation occurs at a lower potential when using the GCE/[Zn(TMPP)(4,4′-bpy)] modified electrode, indicating an enhancement in electron transfer kinetics. This effect can be attributed to the Zn(TMPP)(4,4′-bpy) complex, which likely improves electron mediation, increases the electroactive surface area, and enhances dopamine adsorption on the electrode surface. 5 5 55^53^ The catalytic role of the Zn–based complex may facilitate a more efficient electron transfer pathway by reducing the activation energy for dopamine oxidation.^54^ The negative shift in oxidation potential observed in Fig. 13 provides strong evidence for this improved electrochemical performance, reinforcing the potential of the GCE/[Zn(TMPP)(4,4′-bpy)] electrode as an effective platform for dopamine sensing applications.^55^

#### 3.10.3. Optimization of the experimental parameters

**Fig. 14:**
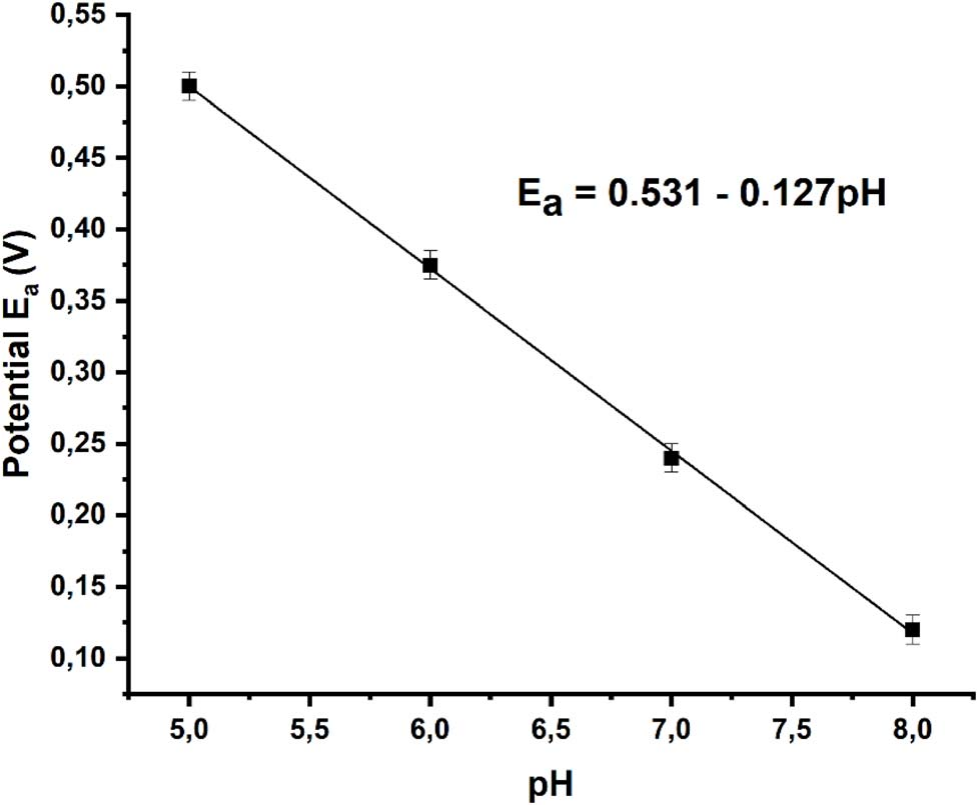
Anodic peak potentials against pH in the presence of 1 mM DA.

To maximize the sensor matrix's sensitivity to dopamine, the effects of several parameters, including drop volume (μL), drying time, electrolyte pH, and incubation time, were evaluated. The optimal conditions for these experimental variables were determined as follows:

- Effect of drop volume (μL): The relationship between current response and drop volume was investigated by applying varying amounts of [Zn(TMPP)(4,4′-bpy)]·CHCl_3_ film suspension (ranging from 5 to 20 μL) onto the GCE. As shown in Fig. S20,† the current response to dopamine (DA) increased with volumes from 5 μL to 7 μL, but further increases in volume led to a suppression of the current. Therefore, 7 μL was selected as the optimal drop volume. This behavior can likely be attributed to the thickening of the composite layer on the GCE surface, which diminishes the electrical conductivity of the modified film.

- Effect of drying time: drying time is a crucial factor that influences both the detection limit and sensitivity of the sensor membrane. To optimize this parameter, the effect of drying time was assessed using a fixed dopamine concentration (10^−3^ M) over a range from 30 minutes to 24 hours, as shown in Fig. S21.† A significant increase in current was observed as the drying time was extended from 30 minutes to 2 hours. However, beyond 2 hours, the current increase slowed, likely due to rapid surface saturation. As a result, 2 hours was selected as the optimal drying time.

- Effect of pH: the effect of pH on the sensor's electrochemical response was examined in the pH range from 4 to 8 using CV and SWV techniques. As shown in Fig. 14, the anodic peak potentials for dopamine (DA) shift negatively with increasing pH. The anodic peak currents increase as the pH rises up to 7, after which a slight decrease is observed. Therefore, a 0.1 M phosphate buffer solution (PBS) with a pH of 7 was selected for further studies. Additionally, the relationship between pH and anodic peak potential was analyzed. Fig. S22† illustrates a clear linear correlation between the anodic peak potentials (*E*_a_) of DA and the pH values within the range of 5 to 8. The linear regression equation for DA is expressed as *E*_a_ (DA) = −0.127 pH + 0.531.

- Effect of incubation time: The interaction between the matrix and dopamine (DA) is influenced by the incubation time, which can affect the electrochemical performance. To optimize this, the accumulation time was varied between 5 and 20 minutes. As shown in Fig. S23,† the optimal accumulation time was found to be around 5 minutes. The optimal experimental conditions were determined to be a drop volume of 7 μL, a drying time of 2 hours, a pH of 7, and a DA incubation time of 5 minutes.

#### 3.10.4. Electrochemical detection of dopamine by GCE/[Zn(TMPP)(4,4′-bpy)]·CHCl_3_

**Fig. 15:**
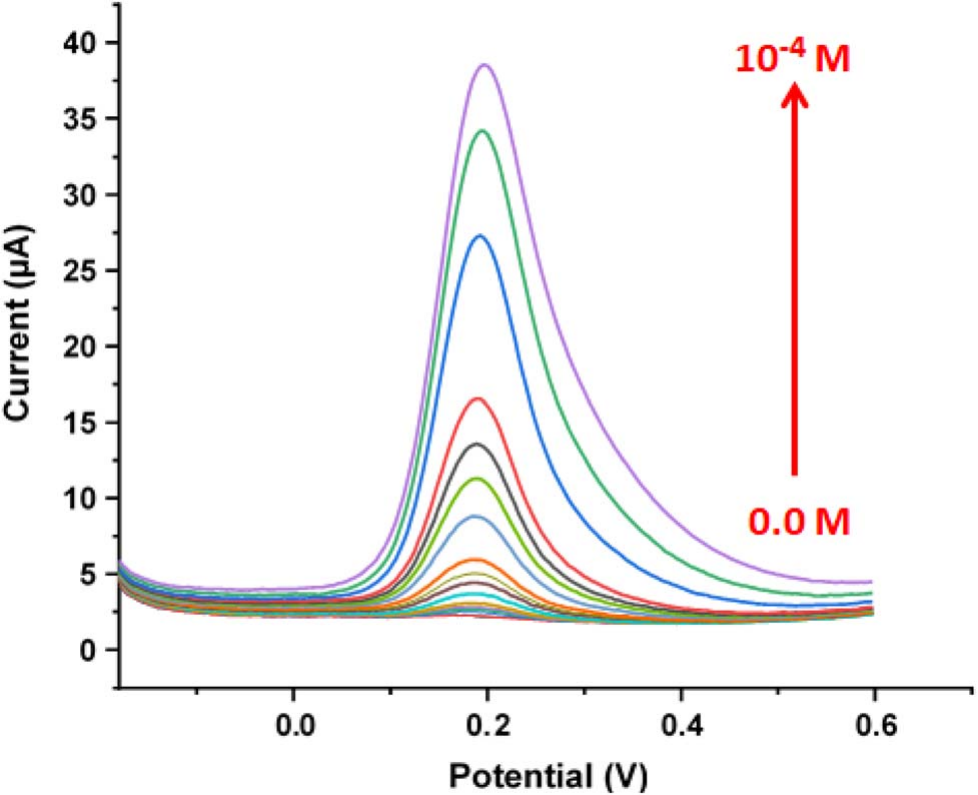
Anodic square wave voltammograms of the modified GCE recorded in the presence of dopamine in 0.1 M PBS at pH 7, with an amplitude of 0.04 V, a step amplitude of 0.004 V, and a frequency of 10 Hz.

**Fig. 16:**
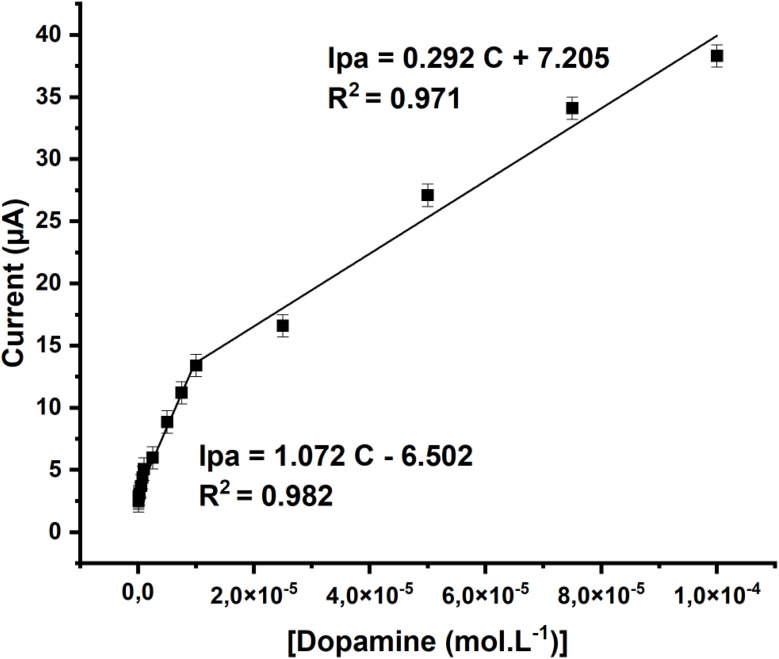
Plot of oxidation peak currents *versus* dopamine concentrations from 0 to 10^−4^ M.

**Scheme 3:**
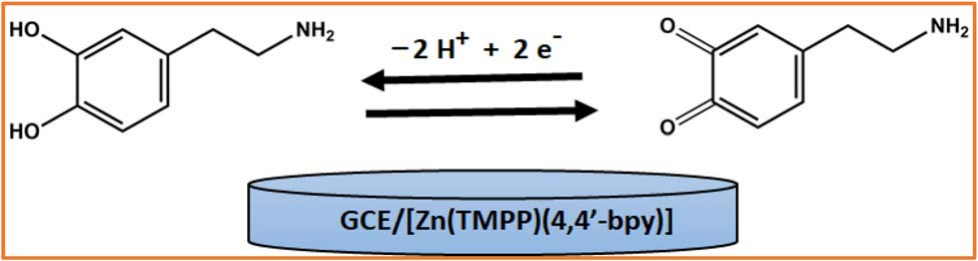
The electroanalytical oxidation reaction of dopamine at the modified electrode GCE/[Zn(TMPP)(4,4′-bpy)]·CHCl_3_.

To assess the electrode's performance for dopamine sensing, square wave voltammetry (SWV)^56^ was conducted using the GCE/[Zn(TMPP)(4,4′-bpy)]·CHCl_3_ modified electrode. As shown in Fig. 15, the characteristic peak current for dopamine in 0.1 M PBS increases with the concentration, ranging from 5.10^−8^ M to 10^−4^ M. Fig. 16 illustrates that the oxidation peak currents improve with increasing dopamine concentrations. The detection limit for dopamine is determined to be 5 × 10^−8^ M, and the electrode's sensitivity is 1.072 μA mol^−1^ L. A comparison of various dopamine sensors is summarized in Table 9. Compared to other film-based dopamine sensors, the GCE/[Zn(TMPP)(4,4′-bpy)]·CHCl_3_ demonstrates superior sensitivity and a low detection limit. Additionally, when compared to standard methods, such as colorimetric techniques, our sensor shows an excellent response. This outstanding electrochemical performance is closely linked to the unique properties of the Zn-porphyrin complexes, which enhance the sensor's functionality. The electrochemical oxidation mechanism of dopamine on the GCE/[Zn(TMPP)(4,4′-bpy]·CHCl_3_ electrode is illustrated in (Scheme 3).^60,61^ Zn-porphyrins facilitate the oxidation of dopamine at the electrode surface by enhancing electron transfer, leading to the formation of dopamine quinone. Their extended π-conjugated system promotes interactions with analytes like dopamine, while the Zn metal center plays a crucial role in modulating redox activity. The porphyrin framework is also integral to modulating electrochemical properties. In particular, *meso*-substituents modify the macrocycle's electronic environment, thereby enhancing its interaction with the electrode and improving conductivity.^62^ Furthermore, the presence of different axial ligands (*e.g.*, 4,4′-bpy) influences their electron-donating and withdrawing properties, thereby influencing both electrochemical and photocatalytic efficiencies.

**Table 9:** Comparison of the modified GCE with other sensitive electrodes used for dopamine detection

Electrode	Method	Detection limit (M)	Linear range (M)	Reference
MIP-GCE/TiO_2_/(PPY-CTS)^a^^,^^b^	DPV	2.81 × 10^−7^ M	10^−6^–10^−5^ M	57
GCE/EG-Ni-Au(NPs)^c^^,^^d^^,^^e^	SWV	10^−7^ M	2 × 10^−7^–10^−4^ M	50
Aggregation of AuNPs induced by copper ions	Colorimetric	2 × 10^−7^ M	5 × 10^−7^–10^−6^ M	58
GCE/rGO/AuNPs^f^	DPV	2 × 10^−5^ M	10^−6–^6 × 10^−5^ M	59
NiO–CuO/GR/GCE^g^	SWV	1.67 × 10^−7^ M	5 × 10^−7^–2 × 10^−5^ M	62
Complex I	SWV	5 × 10^−8^ M	5 × 10^−8^–10^−4^ M	t. w.

a MIP = molecularly Imprinted Polymer.

b PPY-CTS = polypyrrole-chitosan composites.

c EG = electrodeposited graphene oxide.

d GCE = glassy carbon electrode.

e Au(NPS) = gold nanoparticles.

f rGO = reduced graphene oxide.

g GR = graphene.

#### 3.10.5. Dopamine detection in human beings

Dopamine (DA) analysis was carried out using human urine samples. To assess the practical applicability and reliability of our sensor, a known amount of dopamine was spiked into the real samples, and the results are presented in Table 10. The relative recovery was calculated using eqn (8), where CF represents the concentration found in the spiked sample, CU is the concentration found in the unspiked sample, and CA is the concentration of the added analyte.^63^8
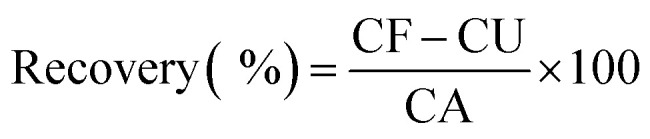


**Table 10:** Application of our sensor for detecting dopamine in human urine samples

Sample	Added dopamine (M)	Found dopamine (M)	Recovery (%)	RSD (%)
Urine	10^−6^ M	0.98 × 10^−6^ M	98	0.970
	5 × 10^−6^ M	5.03 × 10^−6^ M	100.6	1.179
	10^−5^ M	9.75 × 10^−6^ M	97.5	1.032

The significant detection and recovery results show that our modified GCE is promising for the determination of DA in urine human urine samples.

## 4 Conclusion

The [Zn(TMPP)(4,4′-bpy)]·CHCl_3_ coordination compound (complex I), where TMPP is *meso*-tetra(*para*-methoxyphenyl)porphyrinate and 4,4′-bpy is 4,4′-bipyridine, was characterized using UV-Vis spectroscopy, fluorescence, IR, ^1^H NMR, cyclic voltammetry, and ESI-HRMS techniques. X-ray crystallographic analysis revealed that complex I contains a single 4,4′-bpy axial ligand coordinated to the Zn(ii) ion. Additionally, the molecular packing is stabilized by various intermolecular interactions, including C–H⋯Cl, C–H⋯O, C–H⋯N, and C–H⋯Cg (where Cg represents the centroid of a pyrrole, phenyl, or pyridyl ring), involving both the [Zn(TMPP)(4,4′-bpy)] complexes and chloroform solvent molecules. Photocatalytic degradation and H_2_O_2_ oxidative degradation of MB dye using complex I as a catalyst, under optimized conditions, achieved degradation yields of 63% and 53%, respectively. Complex I was further evaluated as an electrochemical sensor for dopamine (DA) detection *via* square wave voltammetry (SWV). The GCE/[Zn(TMPP)(4,4′-bpy)]·CHCl_3_ electrode demonstrated excellent sensitivity (1.072 μA mol^−1^ L) and a low detection limit (5 × 10^−8^ M). Application of this sensor to human urine samples for dopamine analysis highlighted its potential as a reliable electrochemical tool for quantifying dopamine in biological systems.

## Ethical statement

Human urine samples were collected from voluntary donor after obtaining informed consent, following ethical guidelines.

## Data availability

Data are contained within the article.

## Author contributions

Conceptualization, M. A. B., C. M., E. A. L.-M and B. G.; methodology, M. A. B., B. G. and H. N.; validation, M. A. B, C. M., B. G. and H. N.; formal analysis, M. A. B., C. M. and E. A. L.-M; investigation, M. A. B., C. M., F. L., H. B. and H. N.; data curation, M. A. B., T. R. and F. M.; resources, H. N.; writing—original draft preparation, M. A. B, C. M. and H. N.; writing—review and editing, M. A. B., E. A. L.-M and H. N.; investigation, visualitation, A. S-. M. and J. C.-B.; visualization, E. A. L.-M, M. A. B. and H. N.; supervision, H. N. All authors have read and agreed to the published version of the manuscript.

## Conflicts of interest

The authors declare no competing financial interest.

## Supplementary Material

RA-015-D5RA00762C-s001

RA-015-D5RA00762C-s002
