# Supplementary material for: New zinc(ii) metalloporphyrin: molecular structure, spectroscopic characterization, electrochemical sensing of dopamine, and catalytic dye degradation
Source: RSC Adv. 2025 Apr 1;15(13):9810–27. doi: 10.1039/d5ra00762c (PMC11960623; doi:10.1039/d5ra00762c)
Supplement: RA-015-D5RA00762C-s001 [file RA-015-D5RA00762C-s001.pdf]

Supplementary Information (SI) for RSC Advances.
This journal is © The Royal Society of Chemistry 2025

# Supplementary Material of

# New Zinc(II) Metalloporphyrin: Molecular Structure, Spectroscopic characterization, Electrochemical Sensing of Dopamine, and Catalytic Dye Degradation

*Mohamed Achraf Bouicha [a], Chama Mabrouk [b], Bouzid Gassoumi [b], Houcine Barhoumi [b], Florian Molton [c], Frédérique Loiseau [c], Thierry Roisnel [d], Aracely Serrano Medina[e], Jose Manuel Cornejo Bravo[f], Eduardo Alberto Lopez-Maldonad [f]*, Habib Nasri [a,]**

[a] : *University of Monastir, Laboratory of Physical Chemistry of Materials (LR01ES19), Faculty of Sciences of Monastir, Avenue de l'environnement, 5019 Monastir, Tunisia.*

[b]: *University of Monastir, Laboratory of Interfaces and Advanced Materials, Faculty of Sciences of Monastir, Avenue de l'environnement, 5019 Monastir, Tunisia.*

[c]: *Département de Chimie Moléculaire, 301 rue de la Chimie, Université Grenoble Alpes, CS 40700, 38058 Grenoble, Cedex 9, France*

[d]: *Institute of Chemical Sciences of Rennes, UMR 6226, University of Rennes 1, Beaulieu Campus, 35042 Rennes, France*

[e] : *Facultad de Medicina y Psicología, Universidad Autónoma de Baja California, Tijuana 22390, Mexico*

[f] : *Faculty of Chemical Sciences and Engineering, Autonomous University of Baja California, 22424, Mexico.*

**Contents**

**1. Introduction.. ..........................................................................................2**

**2. Experimental Section.. ..................................................................................2**

**3. Mass spectrometry ........................................................................................4**

**4. $^{1}$H NMR spectroscopy ..................................................................................5**

**5. IR spectroscopy ..............................................................................................7**

**6. UV/Vis spectroscopy ......................................................................................8**

**7. Cyclic voltammetry .........................................................................................9**

**8. X-ray Molecular structure .............................................................................10**

**9. Hirshfeld surface analysis ..............................................................................12**

**10. Catalytic photodegradation of methylene blue ...........................................13**

**11. Catalytic Oxidative degradation of MB dye ...............................................14**

**12. Electrochemical sensor application of [Zn(TMPP)(4,4'-bpy)]•$CHCl_3$ (I) ..................15**

## 1. Introduction

Table S1. Number of hits of reported metalloporphyrin crystal structures according to the nature of the metal ion. Data reported from the CCDC Cambridge Structural Database updated to November 2023.

| Metal | Number of hits |
|---|---|
| Zn | 1461 |
| Fe | 1081 |
| Ni | 1002 |
| Co | 393 |
| Mn | 326 |
| Cu | 300 |
| Ru | 279 |

## 2. Experimental Section

### 2.1. Materials and methods

All chemicals used in this study were commercially available and were utilized as received, without additional purification. The *meso*-tetrakis(para-methoxyphenyl)porphyrin ($H_2$TMPP) was prepared via the Adler-Longo protocol. The starting material [Zn(TMPP)] was synthesized according to a previously reported method utilized were readily obtainable commercially and were employed without further purification.

**UV/Vis, FT-IR and NMR data:** A WinASPECT PLUS scanning spectrophotometer (SPECORD PLUS version 4.2 validation) was used to record UV-visible absorption spectra. IR spectra with Fourier transformation were acquired employing a PerkinElmer Spectrum Two FT-IR spectrometer. $^1$H NMR spectroscopy was performed on a Bruker DPX 400 spectrometer and chemical shifts are reported in ppm below the internal tetramethylsilane (TMS) field.

**Fluorescence data:** Emission spectra were obtained at room temperature in dichloromethane using a Horiba Scientific Fluoromax-4 spectrofluorometer, with samples placed in quartz cuvettes of 1 cm path length. Luminescence lifetime measurements were performed following excitation at $\lambda$ = 420 nm for $H_2$TMPP and ZnTMPP, and $\lambda$ = 430 nm for [Zn(TMPP)(4,4'-bpy)]•$CHCl_3$. Excitation was achieved using the second harmonic of a titanium:sapphire laser system (Spectra Physics Tsunami 3950-M1BB with 39868-03 pulse picker doubler) operating at an 800 kHz repetition rate. Decay profiles were recorded with a Fluotime 200 instrument (AMS Technologies)

utilizing a GaAs microchannel plate photomultiplier tube (Hamamatsu R3809U-50) and a time-correlated single photon counting system (PicoQuant PicoHarp 300).

**Electrospray (ESI) MS:** The spectra for the "Electrospray" method, also known as "ESI," were obtained using an amaZon-type instrument. The sample was prepared as a solution in dichloromethane with a concentration of $3.10^{-3}$ M.

**Cyclic voltammetry:** Cyclic voltammetry experiments were conducted at room temperature using a CH-660B potentiostat. The measurements were taken in dichloromethane with a solute concentration of approximately $10^{-3}$ M, using $nBu_4NBF_4$ (0.1 M) as the supporting electrolyte. The experimental setup involved a three-electrode cell, featuring a glassy carbon working electrode, a platinum wire counter electrode, and an Ag/AgCl/KCl reference electrode. Potentials were adjusted to the saturated calomel electrode (SCE) scale using the following equation :

$$E(SCE) = E(Ag/AgCl) + 0{,}047\ V$$

**Catalytic degradation:** Oxidative degradation experiments of MB dye were carried out at room temperature. The procedure involved 5 mg of the catalyst [Zn(TMPP)(4,4'-bpy)]•$CHCl_3$ combined with 10 mL of an aqueous MB dye solution (pH 6). The mixture was stirred at 200 rpm. After the reaction, the solution was filtered, and the dye concentration was determined by recording the absorbance at 664 nm.

**Catalytic photodegradation:** The photocatalytic performance of our catalyst [Zn(TMPP)(4,4'-bpy)]•$CHCl_3$ was studied by decomposing the MB dye under visible light irradiation at room temperature. The photocatalytic reaction was carried out by using 5 mg of the catalyst [Zn(TMPP)(4,4'-bpy)]•$CHCl_3$ and 10 mL of an aqueous solution of MB dye (at pH = 6). Before exposure to irradiation, the suspension mixtures were stirred in the dark for 30 minutes to attain the adsorption–desorption equilibrium of dye molecules on the catalyst surface. The measurement of MB dye concentrations was conducted by observing the changes in the supernatant at 664 nm using a UV/Vis spectrophotometer.

## Electrochemical sensor preparation :

Electrochemical measurements were conducted using an Autolab PGSTAT 302 N (Eco Chemie) controlled by Nova 2.1 software, with all experiments performed at room temperature. The electrochemical cell comprised a three-electrode configuration: a platinum wire as the counter electrode, an Ag/AgCl/KCl reference electrode, and a glassy carbon electrode (GCE) as the working electrode. The employed techniques included cyclic voltammetry (CV), electrochemical impedance spectroscopy (EIS), and square wave voltammetry (SWV). The GCE was cleaned prior

to modification by polishing sequentially with 0.5 µm and 0.03 µm alumina slurries, followed by ultrasonic rinsing in ethanol and distilled water, and then dried with nitrogen gas. A dispersion of 1 mg of [Zn(TMPP)(4,4'-bpy)]•$CHCl_3$ in 1 mL of dichloromethane (DCM) was prepared using ultrasonic agitation to ensure homogeneity. A 7 µL aliquot of this solution was applied onto the GCE surface and allowed to dry under ambient conditions for 2 hours. Electrochemical measurements were performed in a phosphate buffer solution (PBS, 0.1 M, pH 7) as the supporting electrolyte, with the pH adjusted using a JENWAY 3505 pH meter.

### 3. Mass spectrometry

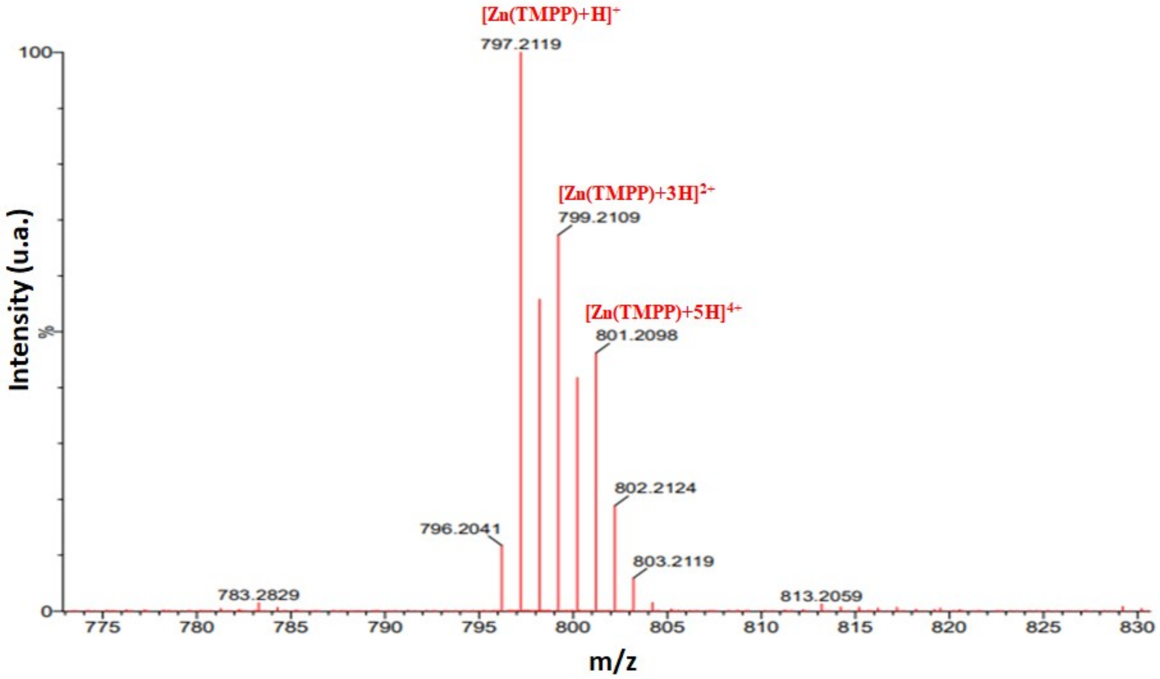


Figure S1. ESI-HRMS full spectrum of [Zn(TMPP)] reordered in dichloromethane with concentration of ~$10^{-3}$ M and solution.

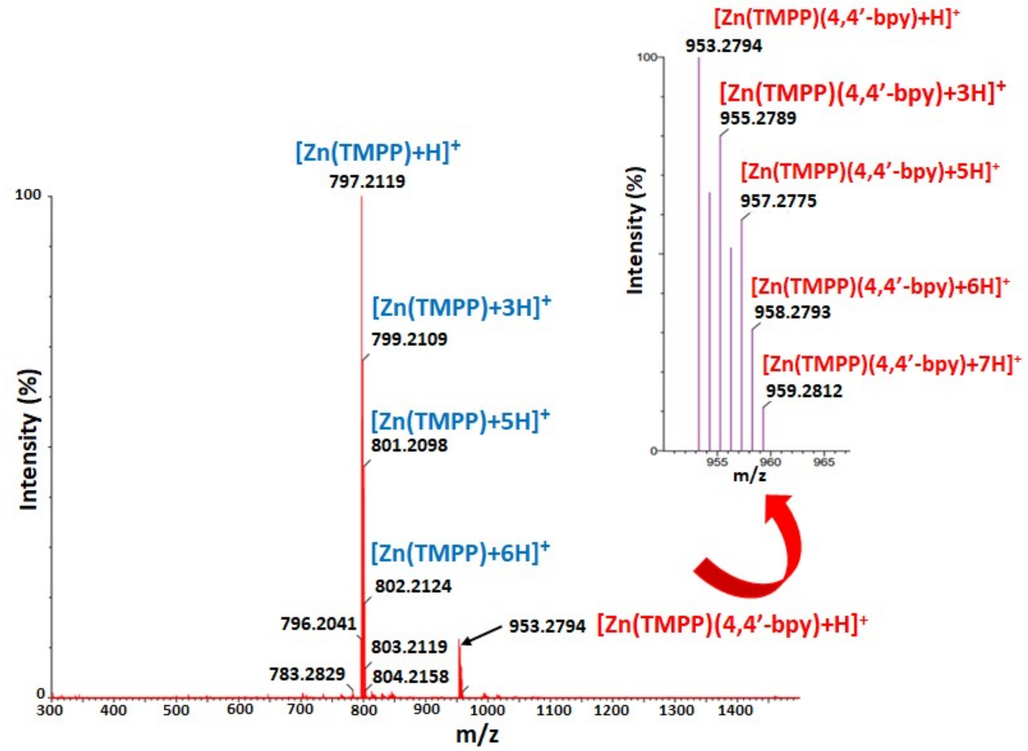


Figure S2. ESI-HRMS full spectrum of [Zn(TMPP)(4,4'-bpy)]·$CHCl_3$.

## 4. $^1$H NMR spectroscopy

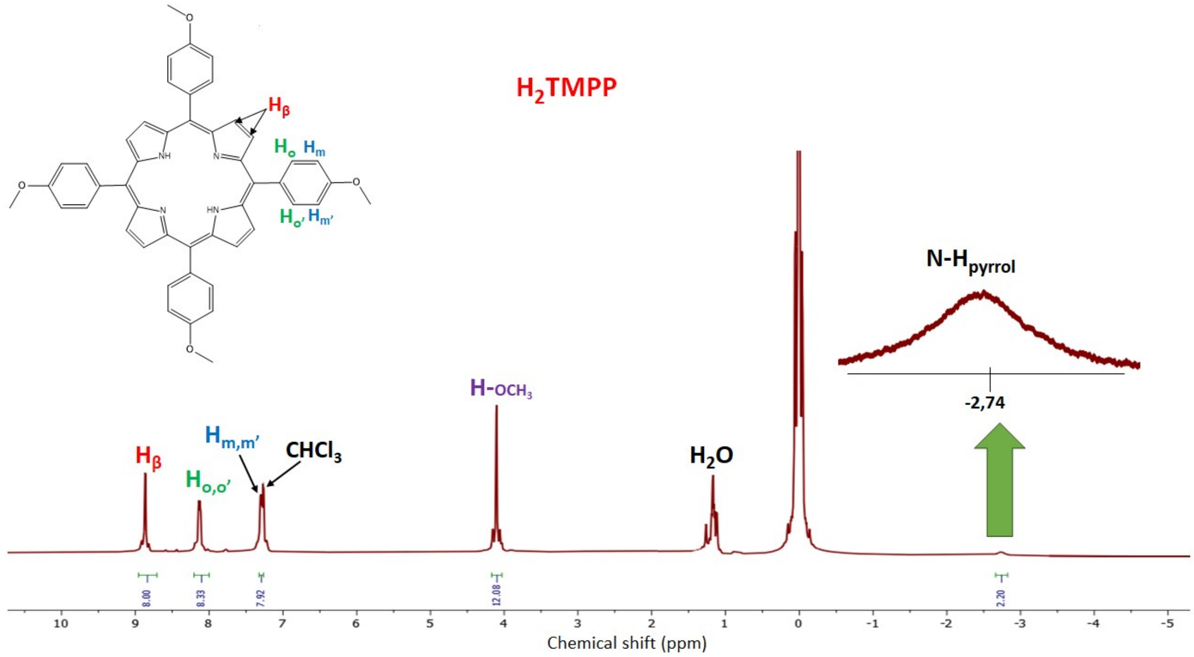


Figure S3. $^1$H NMR (300 MHz) spectra in $CDCl_3$, for the $H_2$TMPP free base porphyrin.

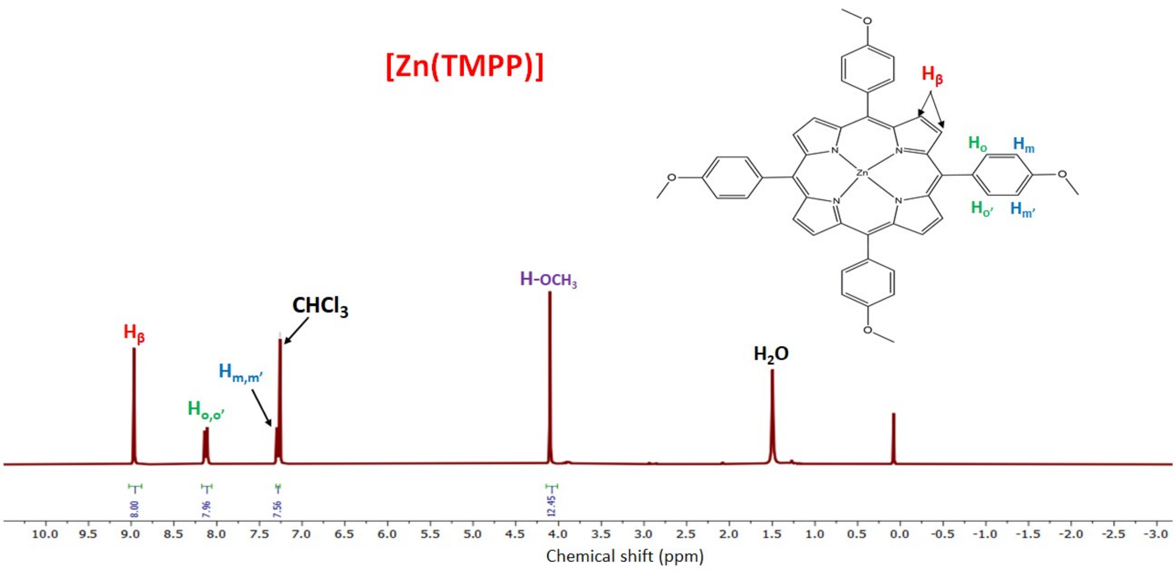


Figure S4. [1]H NMR (300 MHz) spectra in $CDCl_3$, for the [Zn(TMPP)] starting material.

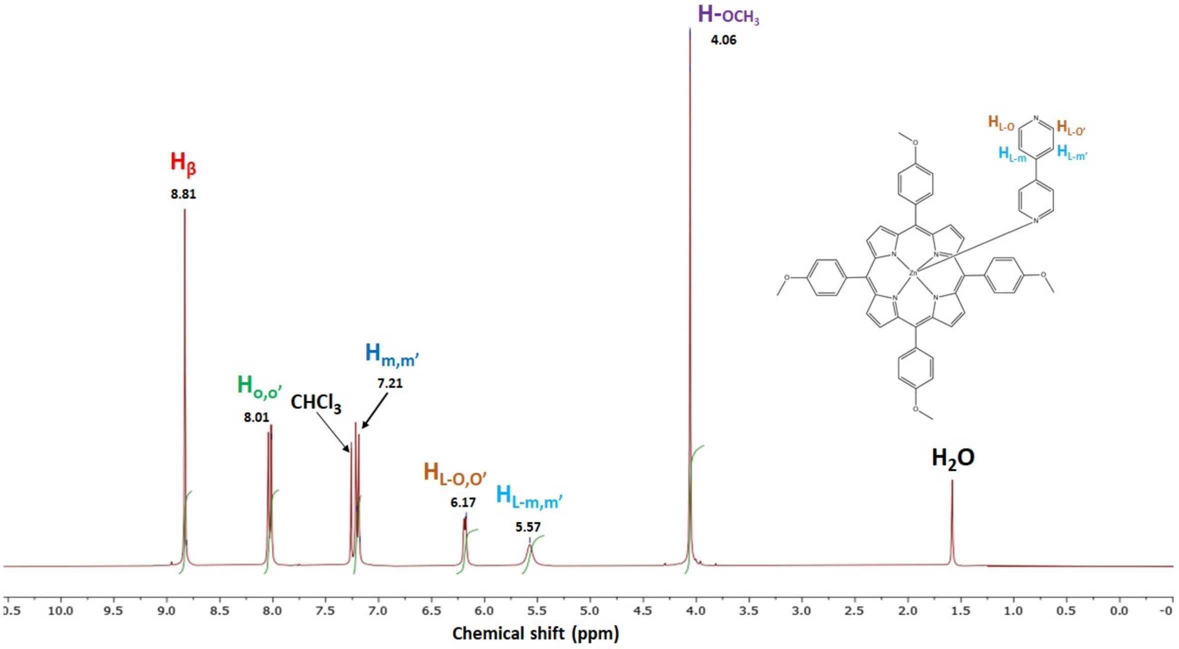


Figure S5. [1]H NMR spectrum of $[Zn(TMPP)(4,4'\text{-bpy})]\cdot CHCl_3$.

Table S2. Chemical shift values for selected free base porphyrins and zinc(II) *meso*-arylporphyrin complexes obtained from $^1H$ NMR spectra recorded in $CDCl_3$ solvent.

| Compound | $H_β$-pyrrolic | H-phenyl | $H\text{-}OCH_3$[a] | NH-pyrrolic | Ref. |
|---|---|---|---|---|---|
| | *Meso-arylporphyrins* | | | | |
| $H_2TTMPP$[b] | 8.95 | 7.48 | 4.12 | -2.75 | **1** |
| $H_2(T_{AzP}\text{-}HVP)$[c] | 8.8 | 7.71-7.53 | 3.93 | -2.68 | **2** |
| $H_2TMPP$ | 8.87 | 8.11-7.29 | 4.11 | -2.74 | t.w |
| | *Zn(II)-meso-porphyrins complexes* | | | | |
| [Zn(TTMPP)][b] | 9.06 | 7.48 | 4.19 | - | **1** |
| [Zn(TMPP)] | 8.95 | 8.14-7.30 | 4.10 | - | t.w |
| $[Zn(T_{AzP}\text{-}HVP)]$[c] | 9.11 | 7.71-7.55 | 3.94 | - | **2** |
| [Zn(TTP)(4,4'-bpy)][d] | 8.84 | 8.04-7.72 | - | - | **3** |
| $[Zn(TMPP)(4,4'\text{-}bpy)]•CHCl_3$ | 8.81 | 8.01-7.21 | 4.06 | - | t.w |

[a]: $H\text{-}OCH_3$= protons of the $OCH_3$ group in the para-phenyl positions of the $H_2TMPP$ porphyrin, [b]: $H_2TTMPP$ : *meso*-tetrakis-(3,4,5-trimethoxyphenyl)porphyrin, [c]: $H_2(T_{AzP}\text{-}HVP)$ = *meso*-tetrakis(3-methoxy-4-((1-phenyl-1H-1,2,3-triazol-4-yl)methoxy)phenyl)porphyrin, [d]: TTP = *meso*-tetra(*p*-tolyl)porphyrin.

## 5. IR spectroscopy

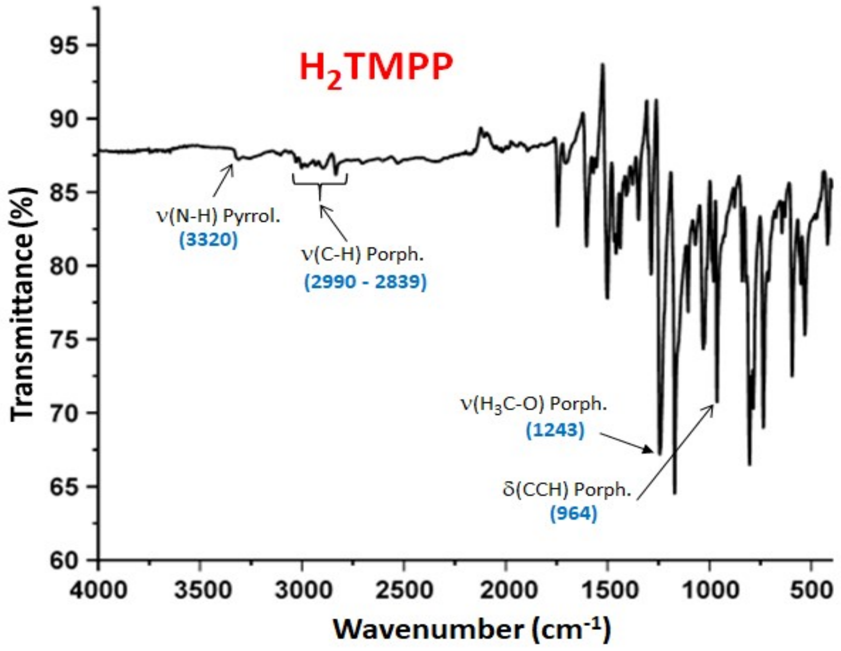


Figure S6. IR spectrum of the free base porphyrin $H_2TMPP$ recorded in solid state in the [4000 - 500] $cm^{-1}$ region..

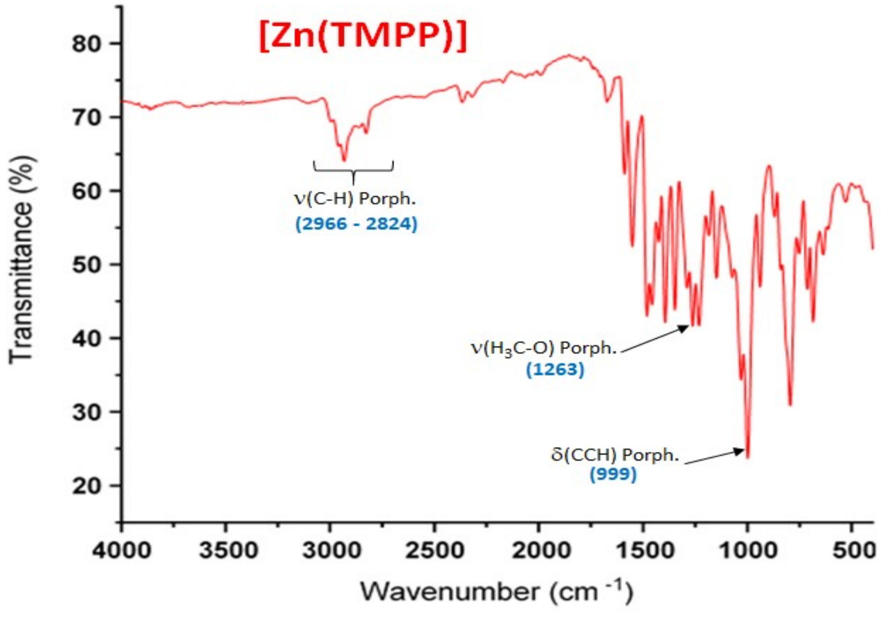


Figure S7. IR spectra of complex [Zn(TMPP)] recorded in solid state in the [4000 - 500] $cm^{-1}$ region.

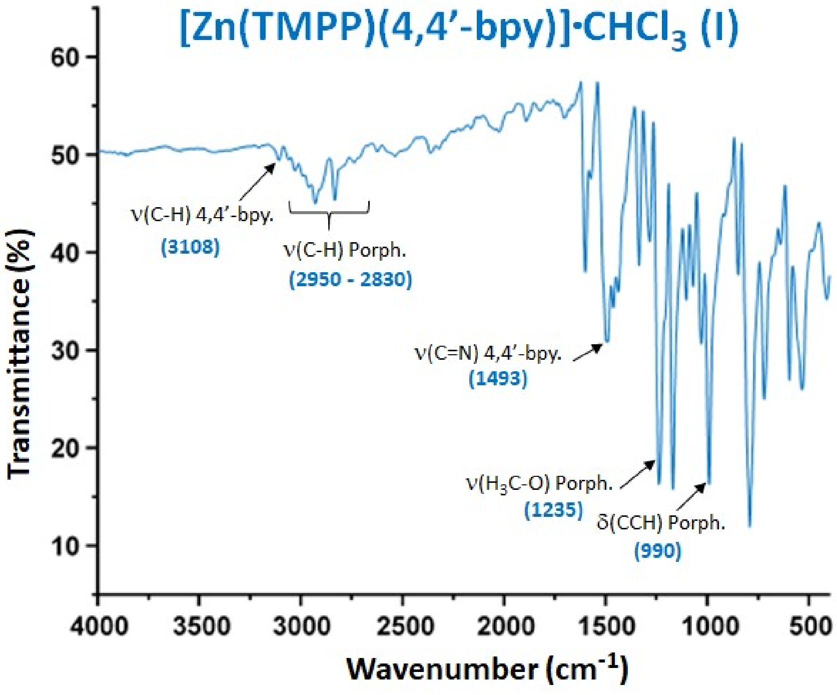


Figure S8. IR spectra of complex [Zn(TMPP)(4,4'-bpy)]•$CHCl_3$ (**I**).

## 6. UV/Vis spectroscopy

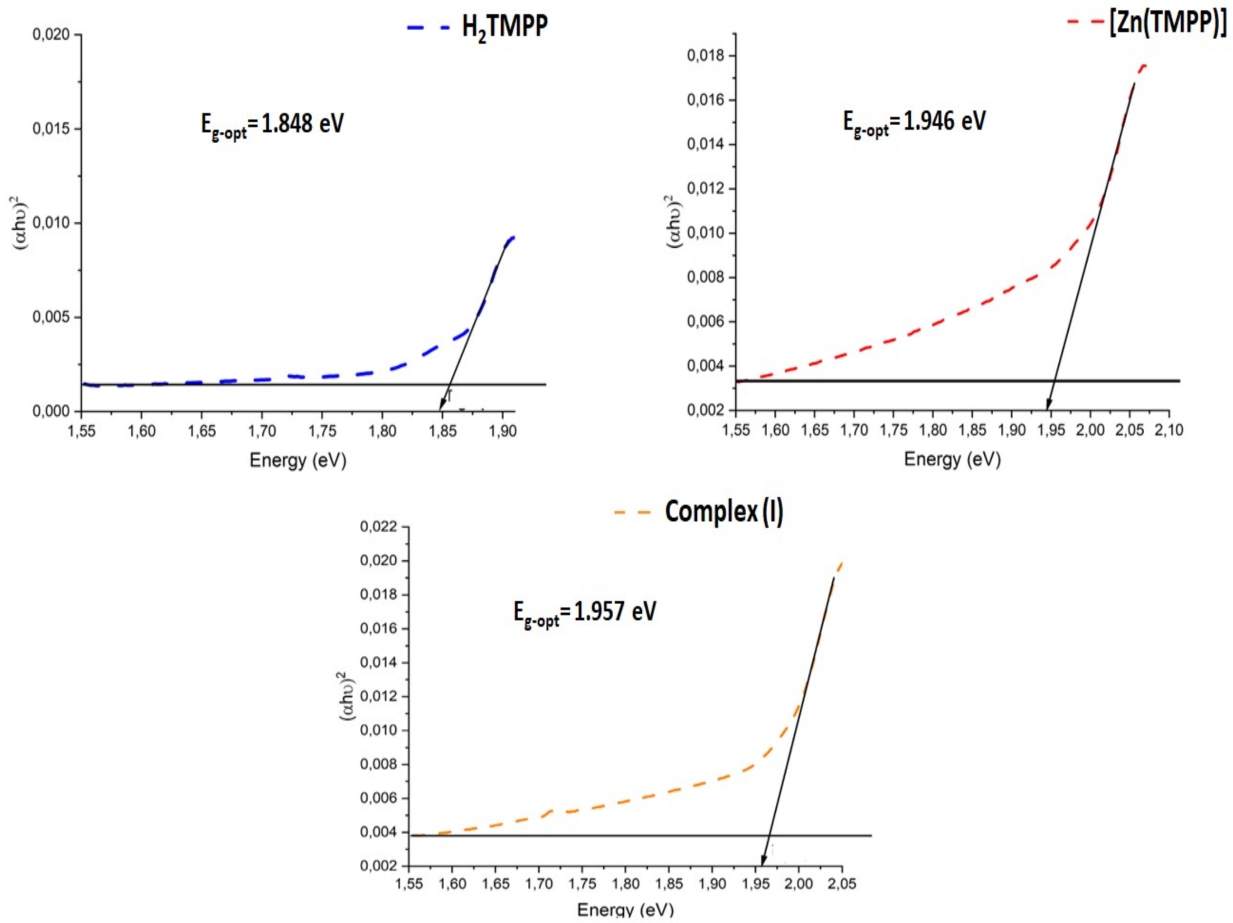


Figure S9. Plots of $(\alpha h\upsilon)^2$ versus the photon energy (hν) of $H_2$TMPP, [Zn(TMPP)] and [Zn(TMPP)(4,4'-bpy)] (**I**). hν is the incident photon energy and α is the absorption coefficient.

## 7. Cyclic voltammetry

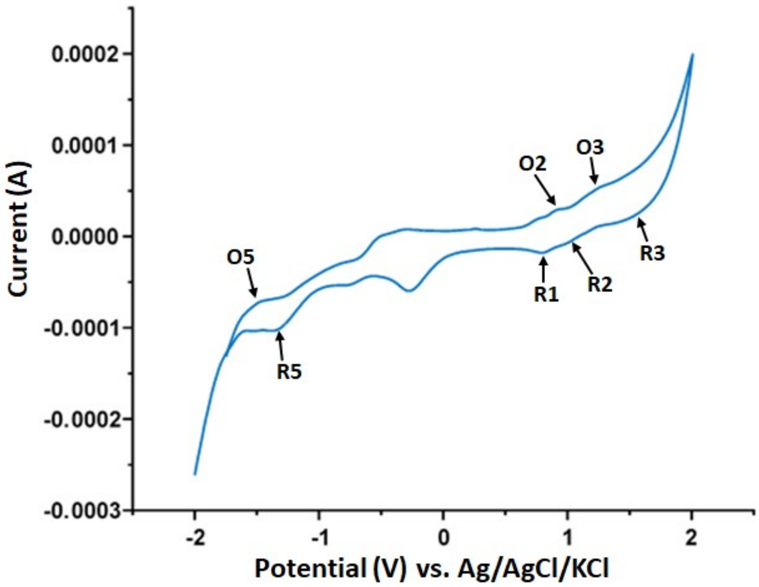


Figure S10. Cyclic voltammograms of [Zn(TMPP)(4,4'-bpy)]·$CHCl_3$ (**I**). The solvent used is the dichloromethane and the concentration is ca. $10^{-3}$ M in 0.1 M $n$$Bu_4NBF_4$, 100 mV/s, glassy carbon working electrode

## 8. X-ray Molecular structure

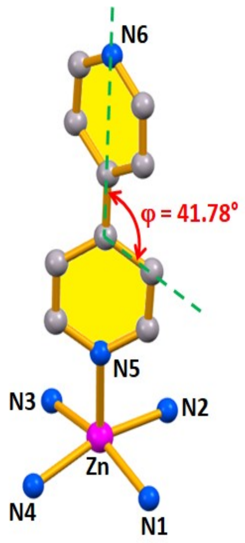


Figure S11. Coordination polyhedron of the zinc(II) center ion in complex **I**.

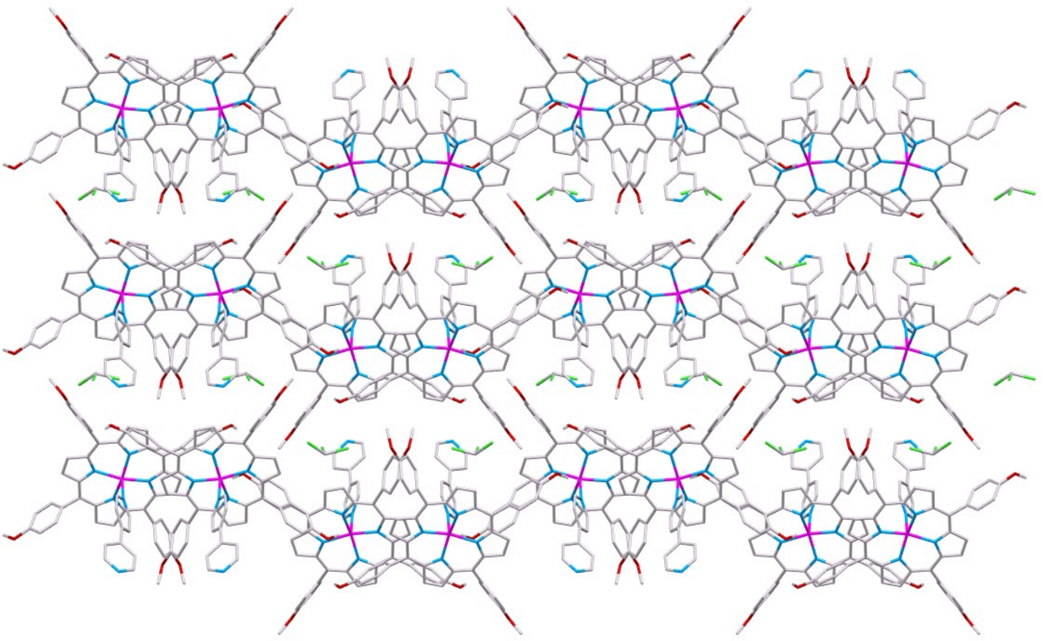

Figure S12. Projection of the crystal lattice of complex **I** down the *b* axis.

Table S3. Selected bond lengths (Å) and angles (°) of **I**.

| | | | |
|---|---|---|---|
| | *Zinc Zn coordination polyhedron* | | |
| Zn-N1 | 2.0794(16) | N2-Zn-N3 | 88.53(6) |
| Zn-N2 | 2.0619(16) | N2-Zn-N4 | 163.01(7) |
| Zn-N3 | 2.0799(16) | N3-Zn-N4 | 88.42(6) |
| Zn-N4 | 2.0517(16) | N5-Zn-N1 | 100.46(6) |
| Zn-N5 | 2.1441(17) | N5-Zn-N2 | 95.05(7) |
| N1-Zn-N2 | 88.62(6) | N5-Zn-N3 | 98.35(6) |
| N1-Zn-N3 | 161.15(6) | N5-Zn-N4 | 101.92(7) |
| N1-Zn-N4 | 88.88(6) | | |
| | *4,4-bipyridine axial ligand* | | |
| N5-C49 | 1.333(3) | N6-C57 | 1.331(3) |
| N5-C53 | 1.332(3) | C57-C58 | 1.390(3) |
| C49-C50 | 1.379(3) | C58-C54 | 1.392(3) |
| C50-C51 | 1.385(3) | Zn-N5-C49 | 119.78(14) |
| C51-C52 | 1.382(3) | Zn-N5-C53 | 123.05(14) |
| C52-C53 | 1.381(3) | C49-N5-C53 | 117.03(18) |
| C51-C54 | 1.482(3) | C51-C54-C55 | 121.33(19) |
| C54-C55 | 1.392(3) | C51-C54-C58 | 121.38(19) |
| C55-C56 | 1.384(3) | C56-N6 | 1.329(3) |

Table-S4. Selected intermolecular interactions for complex **I**.

| D –H....Aa | Symmetry of A | D....A (Å) | D–H...A (°) |
|---|---|---|---|
| C26-H26...Cl1 | -x+1,-y,-z+1 | 3.663(3) | 140 |
| C53-H53...O2 | -x+1,-y,-z+1 | 3.503(3) | 120 |
| C13-H13...Cl3 | -x+1,-y,-z+1 | 3.602(2) | 133 |
| C52-H52...O2 | -x+1,-y,-z+1 | 3.349(3) | 133 |
| C40-H40...O2 | -x+1,-y,-z+1 | 3.470(3) | 140 |
| C56-H56...O1 | x,y,z-1 | 3.511(3) | 121 |
| C57-H57...O3 | x+1,y,z | 3.511(3) | 145 |
| C17-H17...N6 | x-1,y,z | 3.811(3) | 167 |
| C56-H56...O1 | x,y,z-1 | 3.382(3) | 121 |
| C57-H57...O3 | x+1,y,z | 3.511(3) | 145 |
| C17-H17...N6 | x-1,y,z | 3.811(3) | 167 |
| C26-H26...O1 | x,-y+1/2,z+1/2 | 3.405(3) | 135 |
| C43-H43...N1 | x,-y+1/2,z-1/2 | 3.459(3) | 120 |
| C44-H44...N1 | x,-y+1/2,z+1/2 | 3.470(3) | 118 |
| C48-H48...N3 | x,-y+1/2,z+1/2 | 3.747(4) | 145 |
| C41-H41C...O4 | -x,y-1/2,-z+1/2 | 3.798(4) | 143 |
| C46-H46...N6 | x-1,-y+1/2,z+1/2 | 3.702(3) | 147 |
| C3-H3...Cg9 | x,1/2-y,1/2+z | 3.794(2) | 153 |
| C22-H22...Cg1 | x,1/2-y,1/2+z | 3.740(2) | 137 |
| C34-H34A...Cg14 | 1-x,y-1/2,3/2-z | 3.668(3) | 166 |
| C43-H43...Cg1 | x,1/2-y,z-1/2 | 3.403(2) | 143 |

[a] : D = donor atom and A = acceptor atom.
Cg1 is the centroid of the N1/C1-C4 pyrrole ring.
Cg9 is the centroid of the N5/C49-C53 pyridyl ring of the 4,4'-bpy axial ligand.
Cg14 is the centroid of the C42/C43-C47 phenyl ring.

## 9. Hirshfeld surface analysis

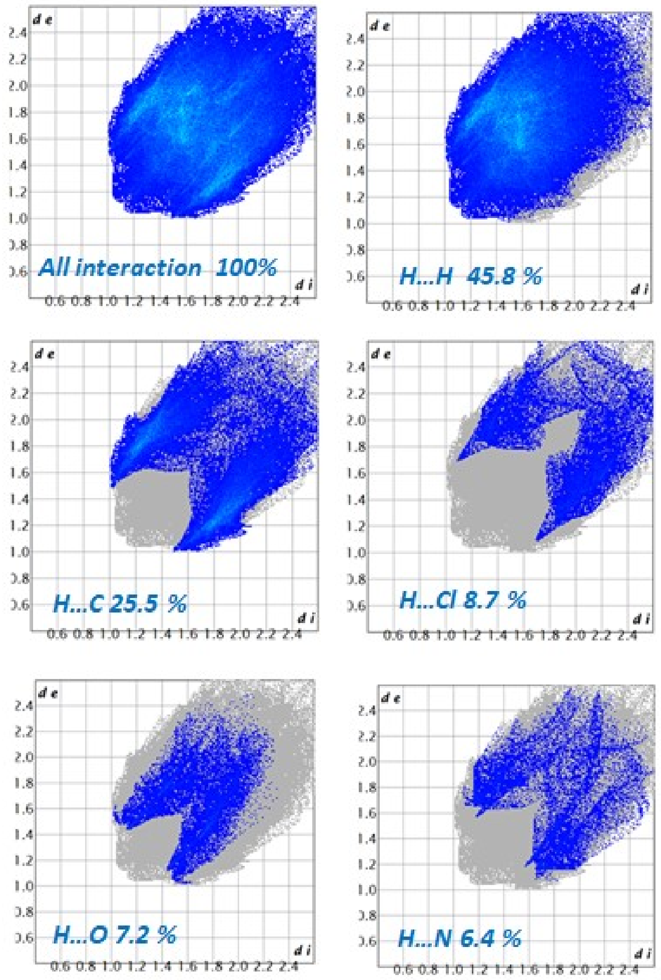


Figure S13. Two-dimensional fingerprint plots for complex **I**

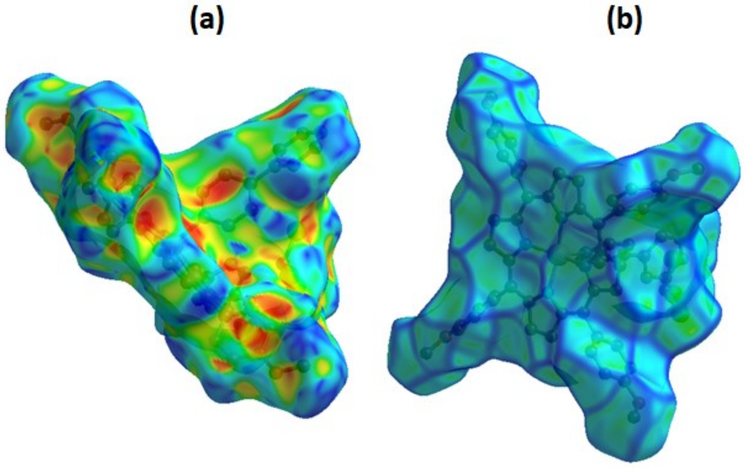


Figure S14. **(a):** Shape index and **(b):** Curvedness of complex **I**.

## 10. Catalytic photodegradation of methylene blue

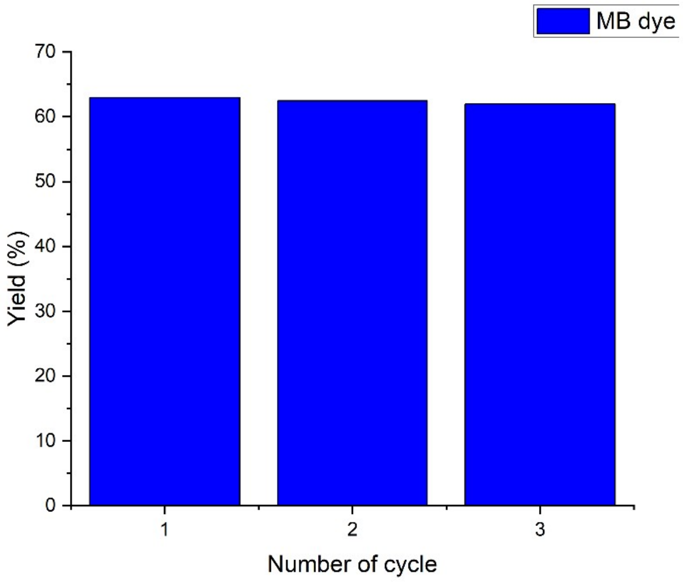


Figure S15. Redegradation efficiency of [Zn(TMPP)(4,4'-bpy)]•$CHCl_3$ (**I**) in the photocatalytic degradation of the MB dye.

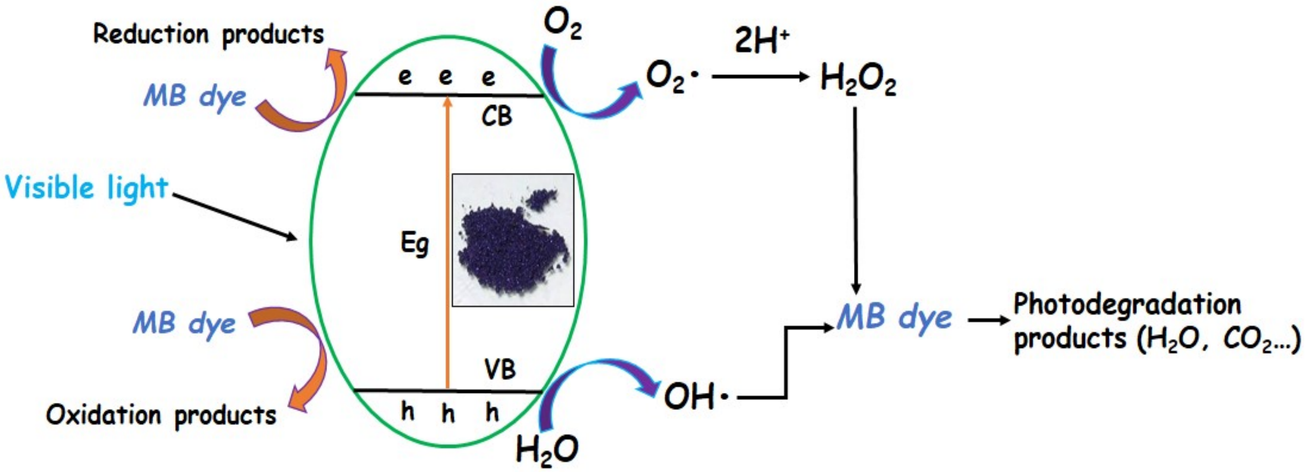


Figure S16. Pictorial representation of indirect dye degradation process.

## 11. Catalytic Oxidative degradation of MB dye

**- Effects of pH:**

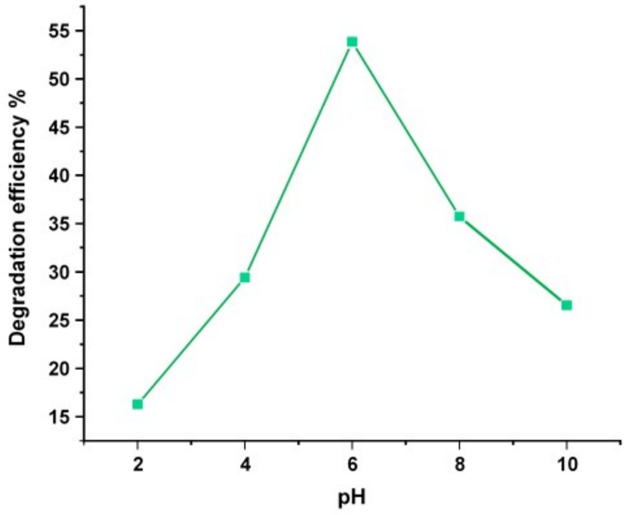


Figure S17. Evolution of the degradation efficiency (%R) of the MB dye versus pH using 5 mg of catlyst. Experiment conditions: Time contact=180 min, T=25 °C, [MB]=20 mg.L$^{-1}$ and [$H_2O_2$]=4 mg.L$^{-1}$.

**- Effects of temperature:**

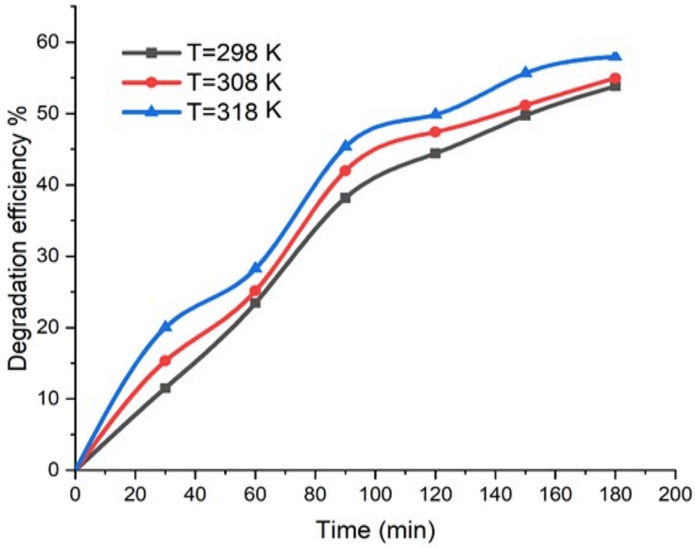


Figure S18. Change in the degradation efficiency of the MB solution over time using complex **I** as catalyst at 298, 308 and 318 K.

## 12. Electrochemical sensor application of [Zn(TMPP)(4,4'-bpy)]•$CHCl_3$ (I)

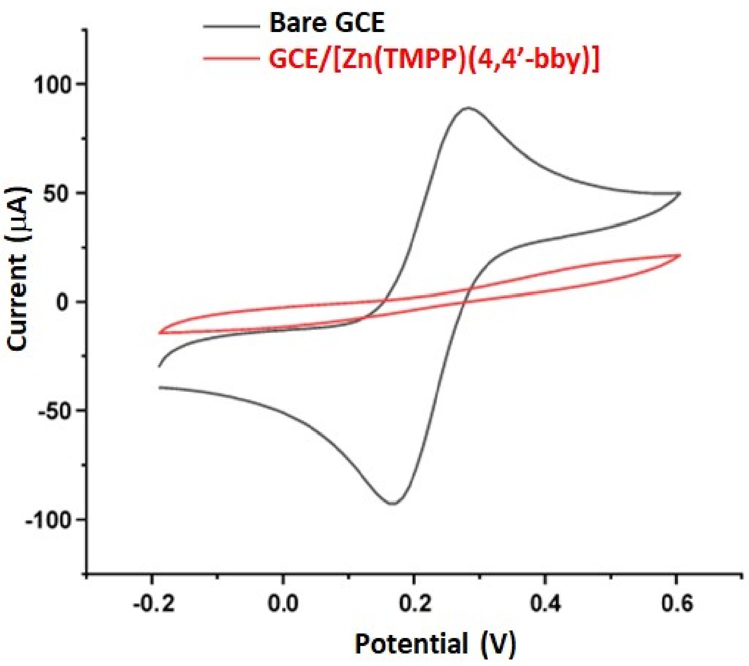


Figure S19. Cyclic voltammograms of 5 mM of $[Fe(CN)_6]^{3-/4-}$ in 0.1 M of KCl of **(a):** bare GCE, **(b):** GCE/[Zn(TMPP)(4,4'-bpy)]•$CHCl_3$ at a scan rate of 50 mV.$s^{-1}$ vs Ag/AgCl/(sat. KCl).

## - Effect of Drop volume (µL):

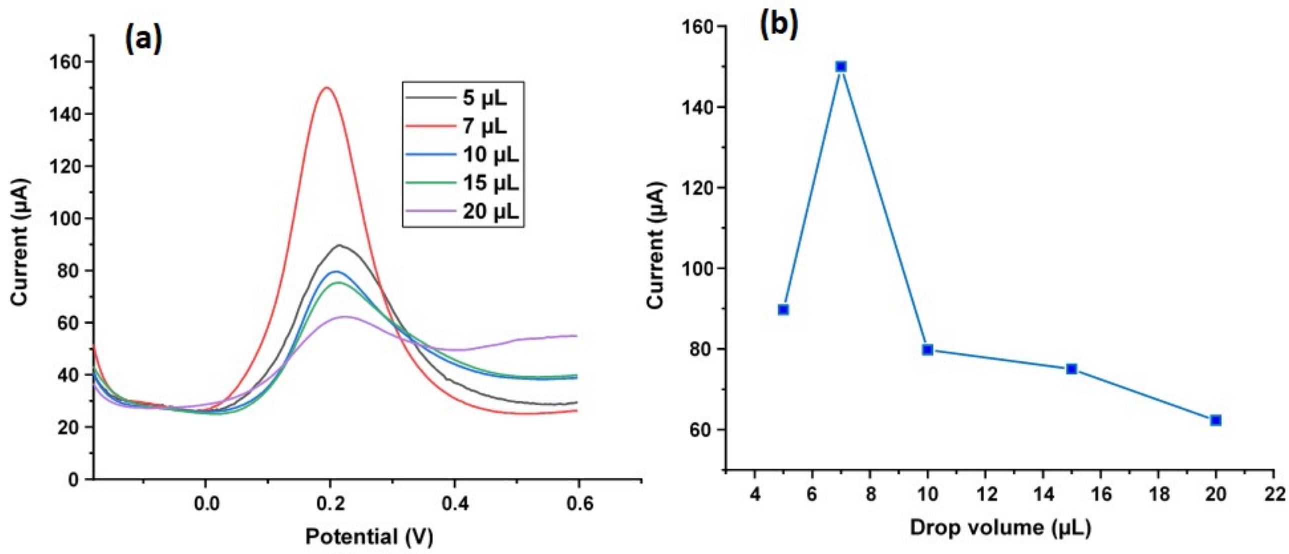


Figure S20. **(a):** Representation of the effect of drop volume (µL), **(b)**: Representation of the relationship between the anodic peak current and the drop volume values in the presence of 1 mM DA.

**- Effect of drying time:**

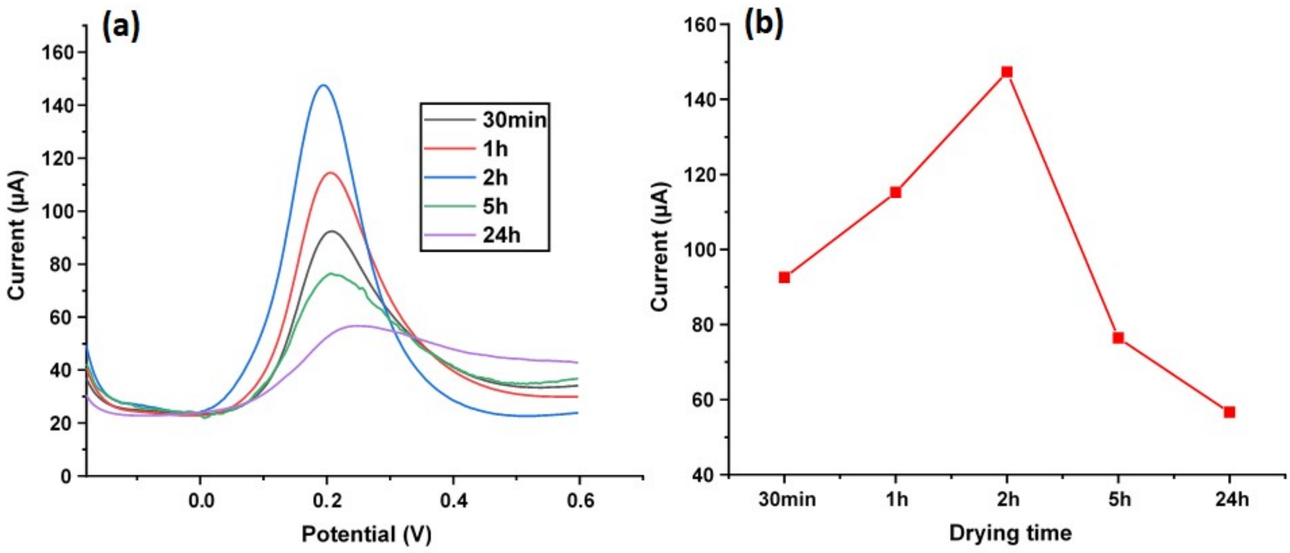


Figure S21. **(a):** Representation of the effect of drying time, **(b):** Representation of the relationship between the anodic peak current and drying time values in the presence of 1 mM DA.

- **Effect of pH:**

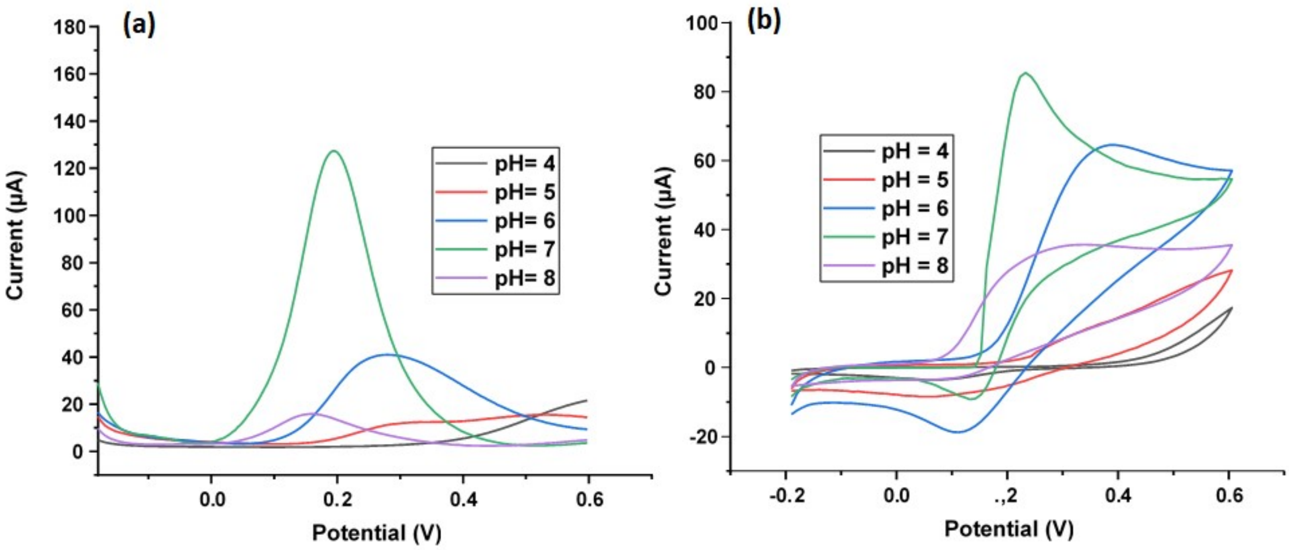


Figure S22. **(a):** Representation SWV, **(b):** Representation CV of dopamine 1 mM in 0.1 M PBS buffer solution at various pHs at GCE modified. The scan rate is 50 mV $s^{-1}$ vs Ag/AgCl/(sat. KCl).

**- Effect of incubation time:**

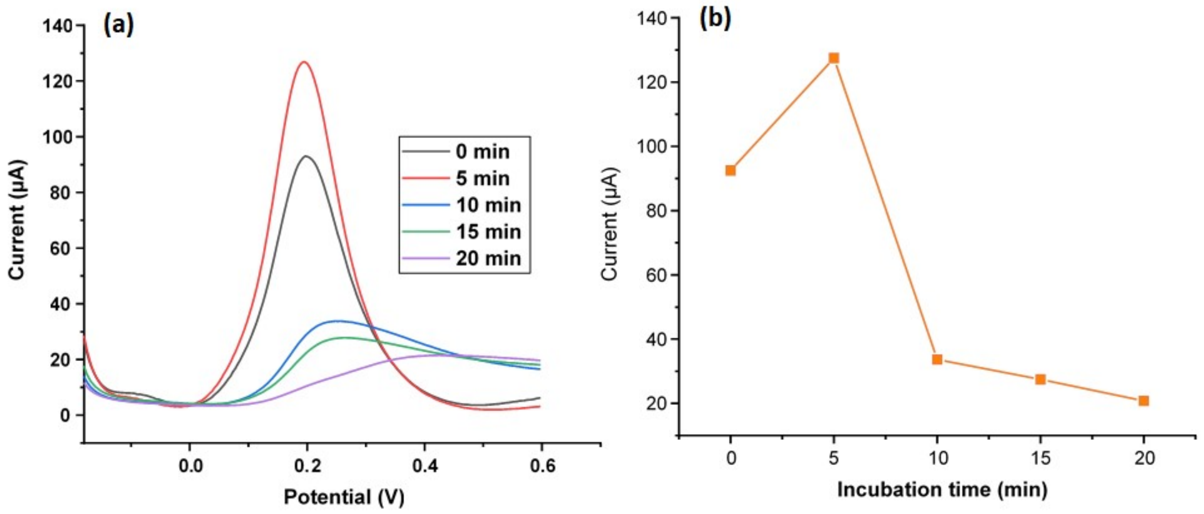


Figure S23. **(a):** The effects of incubation time (min), **(b);** Relationship between the anodic peak current and incubation time values in the presence of 1 mM DA.

## References


**1** R. Soury, M. Chaabene, M. Jabli, T. A. Saleh, R. Ben Chaabane, E. Saint-Aman, F. Loiseau, C. Philouze, A.R. Allouche, H. Nasri, Meso-tetrakis(3,4,5-trimethoxyphenyl)porphyrin derivatives: Synthesis, spectroscopic characterizations and adsorption of $NO_2$, J. Chem. Eng., 2019, 375, 122005. https://doi.org/10.1016/j.cej.2019.122005.

**2** M. Guergueb, J. Brahmi, S. Nasri, F. Loiseau, K. Aouadi, V. Guerineau, H. Nasri, Zinc(II) triazole meso-arylsubstituted porphyrins for UV-visible chloride and bromide detection. Adsorption and catalytic degradation of malachite green dye, RSC Adv., 2020, 10, 22712. https://doi.org/10.1039/D0RA03070H.

**3** R. Soury, M. Jabli, T.A. Saleh, W.S. Abdul-Hassan, E. Saint-Aman, F. Loiseau, C. Philouze, A. Bujacz, H. Nasri, Synthesis of the (4,4′-bipyridine)(5,10,15,20-tetratolylphenylporphyrinato)zinc(II) bis(4,4-bipyridine) disolvate dehydrate and evaluation of its interaction with organic dyes, J. Mol. Liq., 2018, 264, 134-142. https://doi.org/10.1016/j.molliq.2018.05.050.
